# Systematic genetic perturbation reveals principles underpinning robustness of the epigenetic regulatory network

**DOI:** 10.1093/nar/gkaf297

**Published:** 2025-04-17

**Authors:** Thomas Stuart Wilson, Roberta Noberini, Eirini Moysidou, Ifeyinwa Ojukwu, Marta Milan, Ming Jiang, Gavin Kelly, Michael Howell, Tiziana Bonaldi, Paola Scaffidi

**Affiliations:** Cancer Epigenetics , The Francis Crick Institute, London, NW1 1AT, United Kingdom; Nuclear Proteomics, Department of Experimental Oncology, IEO, European Institute of Oncology IRCCS, Milan, 20139, Italy; Cancer Epigenetics, Department of Experimental Oncology, IEO, European Institute of Oncology IRCCS, Milan, 20139, Italy; Cancer Epigenetics , The Francis Crick Institute, London, NW1 1AT, United Kingdom; Cancer Epigenetics , The Francis Crick Institute, London, NW1 1AT, United Kingdom; High-throughput Screening, The Francis Crick Institute, London, NW1 1AT, United Kingdom; Bioinformatics and Biostatistics, The Francis Crick Institute, London, NW1 1AT, United Kingdom; High-throughput Screening, The Francis Crick Institute, London, NW1 1AT, United Kingdom; Nuclear Proteomics, Department of Experimental Oncology, IEO, European Institute of Oncology IRCCS, Milan, 20139, Italy; Department of Oncology and Haemato-Oncology, University of Milano, Milan, 20122, Italy; Cancer Epigenetics , The Francis Crick Institute, London, NW1 1AT, United Kingdom; Cancer Epigenetics, Department of Experimental Oncology, IEO, European Institute of Oncology IRCCS, Milan, 20139, Italy

## Abstract

The molecular control of epigenetic information relies on hundreds of proteins of diverse function, which cooperate in defining chromatin structure and DNA methylation landscapes. While many individual pathways have been characterized, how different classes of epigenetic regulators interact to build a resilient epigenetic regulatory network (ERN) remains poorly understood. Here, we show that most individual regulators are dispensable for somatic cell fitness, and that robustness emerges from multiple layers of functional cooperation and degeneracy among network components. By disrupting 200 epigenetic regulator genes, individually or in combination, we generated network-wide maps of functional interactions for representative regulators. We found that paralogues represent only a first layer of functional compensation within the ERN, with intra- or inter-class interactions buffering the effects of perturbation in a gene-specific manner: while CREBBP cooperates with multiple acetyltransferases to form a subnetwork that ensures robust chromatin acetylation, ARID1A interacts with regulators from across all functional classes. When combined with oncogene activation, the accumulated epigenetic disorder exposes a synthetic fragility and broadly sensitizes ARID1A-deficient cells to further perturbation. Our findings reveal homeostatic mechanisms through which the ERN sustains somatic cell fitness and uncover how the network remodels as the epigenome is progressively deregulated in disease.

## Graphical Abstract

**Graphical Abstract:**
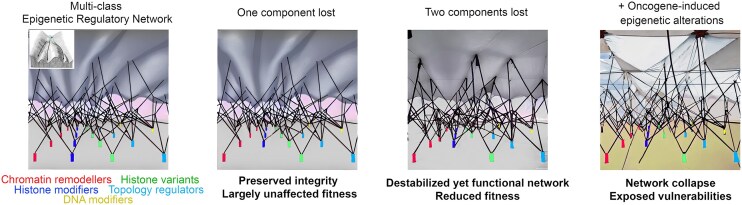

## Introduction

The epigenetic regulatory network (ERN)—the interconnected system of proteins and pathways that govern the establishment, maintenance, and modulation of chromatin and DNA methylation landscapes—controls the functional output of the genome, defining cellular states and behaviors. Reversible covalent modifications of DNA and histones, histone variants, chromatin remodeling, and higher order compaction form multiple regulatory layers that are integrated for the control of gene expression profiles and the maintenance of genome integrity [1, 2]. The identification of writers, readers, and erasers of epigenetic modifications, the biochemical characterization of multi-protein complexes, and the genome-wide mapping of regulators’ binding sites have enabled deep characterization of many pathways [2]. Although efforts have mainly concentrated on studying individual ERN components, initial evidence of regulatory cross talk has emerged, primarily from genetic analysis [3]. Examples include intra-class antagonism between histone methyltransferases of the polycomb and trithorax groups [4], as well as interlayer functional interactions such as the cooperation between the chromatin remodeler SWI/SNF, histone acetyltransferases, and acetylation readers in regulating transcriptional activity [5, 6], or the interplay between the genome topology regulator CTCF and DNA methyltransferases [7, 8]. The combined action of diverse pathways drives emergent system-level properties such as robustness and bistability of ERN outputs, which ensure consistent genome function across environmental and cell-intrinsic fluctuations, or in response to perturbations [9]. Characterizing the regulatory interactions that underpin ERN robustness remains a key goal in understanding how cell identity is controlled and maintained by epigenetic mechanisms. Network topology can be inferred from combinatorial mutagenesis screens, and focused studies in yeast and mammalian cells have begun to expand our repertoire of known interactions [10–12]. Yet, a comprehensive description of how different functional classes within the ERN cooperate in sustaining cell functionality is lacking. Furthermore, studies in human settings have mainly been performed in cancer cell lines or other diseased model systems, where pre-existing chromatin and epigenetic alterations create context-specific confounding effects that make them highly heterogenous and unrepresentative of physiological conditions [13, 14].

Robustness is frequent in biological systems and can emerge through redundancy between duplicated components, observed as the evolution of paralogues with retained overlapping function [15]. Several paralogue pairs exist within the ERN including *ARID1A*/*ARID1B*, *CREBBP*/*EP300*, *KAT2A/KAT2B*, and *SUV39H1/SUV39H2*, and while loss of one paralogue is tolerated—and in fact often selected for in cancer [16]—the combined loss of the gene pair has deleterious effects of varying magnitude, depending on context [13, 17–20]. In addition to highlighting the importance of gene duplication as an evolutionary strategy to increase resilience to perturbation in the ERN, the observed functional compensation between paralogues has also been explored as a therapeutic opportunity for synthetic lethality in cancer; many of these duplicated genes are frequently inactivated across cancer types, and mutated cells are selectively dependent on the functional paralogue, creating cancer-specific vulnerabilities [17, 19–21]. However, these synthetic lethal phenotypes are often lowly penetrant across variable genetic and epigenetic contexts [22], indicating that epistatic interactions influence how cells respond to the loss of a paralogue pair, and that other factors can compensate for their combined absence.

A second level of redundancy is provided by degeneracy, the presence of structurally distinct elements whose function converge on a common output [23]. Degeneracy is particularly prominent in the context of histone modifiers; multiple enzymes share common substrates on histone tails, for instance, several nonparalogous methyltransferases modify H3K36 [24] and diverse COMPASS complexes methylate H3K4 [25]. Furthermore, distinct modifications that induce similar biochemical effects on chromatin and are associated with common functional consequences have evolved, and often co-occupy genomic regions, e.g. multiple acetylated residues decompact chromatin and correlate with transcriptional activity. Degeneracy is also observed as the presence of distinct, but functionally related protein complexes that share some subunits and have both redundant and specific functions. The SWI/SNF chromatin remodeling complexes represent a prototypical example of this form of degeneracy, with 29 protein subunits assembling in various combinations in three complexes (cBAF, PBAF, and ncBAF), which exert partly overlapping functions in the regulation of transcription and genome maintenance [26].

Finally, robustness can emerge as a result of parallel pathways that lead to similar functional consequences via distinct biochemical routes. As a paradigm, gene silencing can be mediated by DNA methylation or heterochromatin formation [27], with multiple biochemically and functionally distinct protein complexes involved (e.g. polycomb repressor complexes PRC1 and PRC2 [28]), as well as adaptor proteins that connect the two pathways of gene repression: UHRF1 binds to repressive histone marks and recruits DNA methyltransferases [29], whereas the PRC1 component KDM2B recognizes CpG islands and recruits the rest of the complex to methylated regions [30].

Redundancy by structural homology, degeneracy, or parallel pathways can be experimentally revealed by epistatic mutant phenotypes [31]. Although examples of each modality have been described, systematic genetic interaction mapping within ERN has not been performed, limiting our knowledge of functional hubs, our understanding of buffering mechanisms upon perturbation, and our ability to predict the extent of tolerance to interference. Identifying functional connections that build a robust ERN has implication for disease, where this robustness is compromised. Epigenetic alterations are common to many pathological conditions: mutations in epigenetic regulator genes (ERGs) play a causal role in various congenital disorders [32], accumulate in ageing somatic cells [33], and contribute to carcinogenesis in multiple cancer types [34]. Furthermore, oncogenic signaling impacts the epigenome, resulting in genome-wide changes in chromatin and DNA methylation patterns in transformed cells, as documented for oncogenic drivers such as KRAS [35, 36], EGFR [37], or MYC [38]. At later stages of cancer development, additional epigenetic alterations superimpose to those acquired during tumor initiation, as a result of interaction with tumor microenvironment and extracellular signaling, and because of subclonal mutations that often target ERGs [16]. Although the progressive accumulation of epigenetic alterations is often selected in cancer, suggesting advantageous functional consequence for malignant cells, the increased regulatory disorder may impact the ability of the network to enact consistent function and create vulnerabilities. Even highly robust networks will collapse under sustained or targeted perturbation, but the threshold for network collapse—how much disorder can be endured—is unknown. Clarifying these principles is important in understanding how a prospective epigenetic therapy could be both tolerated by normal tissues and deleterious to the disrupted network of a tumor. A better understanding of functional connections within the ERN would also enable prediction of synthetic lethal phenotypes in cancers harboring mutations in ERGs.

In this study, we systematically interrogate ERN robustness in somatic cells derived from the normal epithelium of two human tissues, examining the cellular response to individual and combined perturbations. We find that the ERN is remarkably resilient to genetic inactivation of network components in physiological settings, but that accumulated epigenetic disorder in neoplastic cells creates vulnerabilities that may be exploited to interfere with malignant phenotypes.

## Materials and methods

### Generation and culture of Cas9-expressing cells

HCEC-1CT cells were acquired from Evercyte (CHT-039-0229) and cultured in a mix of four parts Dulbecco’s modified Eagle’s medium (DMEM; Gibco) to one part medium 199 (Sigma–Aldrich, M4530) containing 2% cosmic calf serum (GE HyClone, SH30087.02), 4 mM GlutaMAX (Gibco, 35050038), 20 nM hEGF (Sigma–Aldrich, E9644), 100 μM insulin (Sigma–Aldrich, I9278), 2 mg/ml apo-transferrin (Sigma–Aldrich, T2036), 5 nM sodium selenite (Sigma–Aldrich, S5261), and 1 μg/ml hydrocortisone (Sigma–Aldrich, H0396). hTERT-HME1 [ME16C] cells were acquired from ATCC (CRL-4010) and cultured in complete HuMEC medium (Gibco, 12752010) with 2% fetal bovine serum (FBS) (Gibco, 16000-044). All media contained 100 U/ml penicillin and 100 μg/ml streptomycin (Sigma, P4333), and cells were cultured in normoxia at 37°C in 5% CO_2_. To enable efficient knockout of epigenetic regulators, both cell types were transduced with the doxycycline-inducible lentiviral Cas9 vector pCW-Cas9 [39], and high-activity clones that were responsive to 1 μg/ml doxycycline were derived and screened as previously described [16].

### Generation and validation of knockout lines

For generation of monoclonal lines lacking ARID1A or CREBBP, cell populations were pre-treated with doxycycline for 24 h to induce Cas9 expression and transfected with synthetic guide RNAs (gRNAs) targeting *ARID1A* and *CREBBP*. Synthetic gRNAs were formed by complexing CRISPR RNAs (crRNAs) (listed in Supplementary Table S5) with trans-activating CRISPR RNAs (tracrRNAs) (Dharmacon U-002005-20) to form specific gRNAs, and reverse transfecting at 20 nM with 0.15 μl per well of Dharmafect4 (HME1, Dharmacon, T-2004-01) or Lipofectamine 3000 (HCEC-1CT, ThermoFisher, L3000001). Growth medium was replaced after 24 h, and after 72 h individual cells were sorted into multiwell plates using a MoFlo XDP cell sorter. Clonal populations were raised and screened by immunofluorescence detecting ARID1A or CREBBP. CE-DKO cells were generated using a similar strategy, transfecting *EP300*-targeting gRNAs into CREBBP-KO HME1 cells. Sixty clones were isolated and screened by immunofluorescence detecting EP300 and H3K27ac. For genotyping, gDNA was isolated from candidate clones using DNeasy Blood & Tissue Kit (Qiagen, 69504) and the edited loci were amplified by polymerase chain reaction (PCR) using Herculase II Fusion DNA Polymerase (Agilent, 600679) and primers listed in Supplementary Table S5. PCRs were run at 72°C for 5 min, at 98°C for 30 s, 35 cycles of: 98°C for 30 s; 60°C for 20 s; and 72°C for 60s, and then finally at 72°C for 5 min. Amplicons were sent for Sanger sequencing by Source BioScience using the forward primers without the MiSeq adapter sequence TCGTCGGCAGCGTCAGATGTGTATAAGAGACAG. The samples were indexed by PCR using Q5 High-Fidelity DNA Polymerase 2× Master Mix (NEB, M0492L) with custom UDI primers as per the following program: 95°C for 3 min, 10 cycles of: (95°C for 30 s, 55°C for 15 s, and 72°C for 30 s), and then 72°C for 5 min. Indexed libraries were purified with a 1× SPRISelect bead cleanup (Agilent) and the quality and fragment size distributions of the purified libraries were assessed by a 4200 TapeStation Instrument (Agilent Technologies). Libraries were loaded and run on the MiSeq platform (Illumina) in PE 250-bp configuration for an expected 3000–5000 reads per sample, and were analyzed to identify editing outcomes using CRISPREsso2 [40]. To avoid potential clonal effects that would confound the analysis, three–six clones were pooled for each target in each cell line to form the final populations, including a matched pool of parental subclones. For generation of polyclonal populations lacking KMT2D, KAT2A, SUV39H1, HDAC1, and RNF40, pools of specific crRNAs were used following the above protocol and cells were fixed for immunofluorescence microscopy 72 h after guide transfection. Edited, enzyme^low^ cells were defined as those showing fluorescence intensity values lower than the median of the control population.

### Low-pass whole genome sequencing

Nextera Flex libraries were prepared manually following the manufacturer’s protocol (1000000025416, v04) (Illumina), modified to use one-fourth volumes. Sample input was not normalized, and ranged between 20–85 ng of genomic DNA in 7.5 μl. To fragment samples and add adapters, 2.5 μl Tagment buffer 1 and 2.5 μl of bead-linked transposome were mixed with each sample and incubated at 55°C for 15 min. To stop the reaction, 2.5 μl Tagment stop solution was added and the samples incubated at 37°C for 15 min. A post-tagmentation clean-up was performed using Tagmentation wash buffer. For the amplification of the library, 10 μl of enhanced PCR mix (1×) was added, plus 2.5 μl of UDI primers (custom order from IDT). Libraries were amplified by eight PCR cycles (3 min @ 68°C, 3 min @ 98°C, eight cycles of: [45 s @ 98°C, 30 s @ 62°C, and 2 min @ 68°C], then 1 min @ 68°C), followed by 1× bead cleanup with Agilent SPRIselect Beads (B23318; Beckman Coulter). The quality of the purified libraries was assessed using a High Sensitivity D1000 kit on an Agilent Tapestation 4200 Instrument. Libraries were sequenced to a depth of at least 25 M paired-end reads on a HiSeq4000. Copy number estimation was performed using the QDNASeq package [41] following alignment to the Hg38 reference genome.

### Targeted knockout fitness assays

Cell populations were pre-treated with doxycycline for 24 h to induce Cas9 expression. Synthetic gRNAs were formed by complexing crRNAs (listed in Supplementary Table S5) with tracrRNA (Dharmacon U-002005-20) to form specific gRNAs, and reverse transfecting at 20 nM with 0.15 μl per well of Dharmafect4 (HME1, Dharmacon T-2004–01) or Lipofectamine 3000 (HCEC-1CT, ThermoFisher L3000001). Growth medium was replaced after 24 h and every 48 h subsequently, and cells were passaged at 3 and 7 days after transfection. The plates were fixed at endpoint with 4% paraformaldehyde (PFA; Alf Aesar, 43368) and nuclei were stained using SYTOX green (ThermoFisher, #S7020). Whole wells were imaged for quantification using an Incucyte S3 Live-Cell Analysis System.

### Large-scale knockout fitness assay

Two hundred genes encoding *stricto sensu* epigenetic regulators were selected as targets from an available single guide RNA (sgRNA) library [42] after filtering out genes that were unexpressed in either cell line by assessing RNA sequencing (RNA-seq; Supplementary Table S1). The testis-specific ERG *TNP2* was selected as a negative control and confirmed as unexpressed in both cell lines. Targets were arranged on four edge-excluded 96-well plates, so that each plate contained 50 target genes and 10 negative controls (Supplementary Table S1). Lentiviral particles containing the library of pLenti-BSD-sgRNA constructs were generated as previously described [16] with some amendments. Briefly, HEK293T cells were transfected in DMEM supplemented with 10% FBS, and the medium was changed for DMEM supplemented with 2% FBS 12 h after transfection. The supernatant was harvested 36 h after transfection and filtered as described. The lentiviral supernatant was diluted 1:12 in the cell-line specific culture media containing 5 μg/ml polybrene (Santa Cruz Biotechnology, sc-134220) after using a matched GFP-expressing vector to determine the optimal titer. Briefly, 2000 cells per well were seeded in 96-well plates in media containing doxycycline, 24 h prior to infection. Cells were transduced with the lentiviral library and cultured for 11 days. Medium was replaced every 2 days, and the cultures were passaged at 4 and 8 days after infection. The plates were fixed at endpoint with 4% PFA (Alf Aesar, 43368) and nuclei stained using SYTOX green (ThermoFisher, #S7020). Whole wells were imaged for quantification using an Incucyte S3 Live-Cell Analysis System. The fitness score for each gene was determined by log_2_—transforming the cell count for a given gene normalized to the average of negative control values on the plate. ERGs whose knockout led to fitness scores falling within the observed range of negative control values for each cell line were considered fully dispensable, while those that were stronger than the half-maximal effect were considered indispensable (HCEC-1CT: −0.433; for HME1 was taken as −1 as the maximal populations were trending toward eradication). The remaining ERGs were considered partially dispensable. To determine interactors, we used a general linear model on the log-transformed measurements, applied separately to each gene in turn, to estimate and test the association with condition using maximum likelihood and a Wald test, using the mean of the relevant plate’s log-transformed negative control measurements as an offset to account for systematic effects. To do so, we used the linear regression function ∼lm(log_value∼offset(log_plate_TNP2) + Condition, data = .x) in R v4.1.2 [43]. Interactors were considered significant at *P* < 1 × 10^−3^ and abs (regression coefficient) > 0.2.

### Immunofluorescence microscopy

Cultured cells were fixed in 4% PFA and immunostained using standard protocols as previously described [39] using primary and secondary antibodies listed in Supplementary Table S6 at the following dilutions: anti-H3K4me3 (1:350), anti-H3K9ac (1:1100), anti-H3K9me3 (1:2000), anti-H3K27ac (1:200), and anti-H3K120Ub (1:200, following methanol fixation and permeabilization guidelines of manufacturer). Monoclonal knock-out (KO) cell lines were analyzed 24 h after seeding, while polyclonal knockout populations were analyzed 96 h after gRNA transfection. CE-DKO clones were analysed 21 days after *EP300* sgRNA transfection. Plates and slides were imaged on a Cellomics ArrayScan VTI (ThermoFisher), a Nikon Eclipse Ti2, an Axiovert Zeiss confocal and analyzed using HCS Studio (version 6.6.2) or CellProlifer (version 4.2.1) to identify nuclei and measure signal intensity, or an Incucyte SX5 using the Cell-by-Cell Analysis Software Module. All displayed values are indicated in the figure legend.

### Short-term proliferation assays

For comparative proliferation assays, six replicates of each condition were seeded onto 96-well plates at 2000 cells/well, grown for the indicated time in cell culture medium supplemented with 1:1000 SPY650 (Spirochrome, SC501), and imaged at 4–8 h intervals with an Incycte S3 Live-Cell Analysis System. Apoptotic cells were labeled using 5 μM DEVD-NucView-488 (Peptide Chemistry, The Francis Crick Institute) [44] in live culture. Proliferating cells were identified using a 2-h incubation with 105 μM EdU (Invitrogen, C10357A). After fixation with 4% PFA and permeabilization with 0.1% Triton-X, EdU was detected with a biotin-azide click reaction by incubating for 5 min in labeling solution containing 100 mM Tris–HCl (pH 7.6), 4 mM CuSO_4_, 5 μM sulfo-cyanine5 azide (Lumiprobe A3330), and 100 mM sodium ascorbate.

### A-485 treatment

For the proliferation assay, cells were seeded at 2000 or 500 per well and treated with 4 μM A-485 (Cayman Chemical CAY24119) or dimethyl sulfoxide (DMSO) for 72 h with refreshment every 12 h, and confluence was monitored in an Incucyte S3 system. For other treatments, cells were treated with 4 μM A-485 1–2 h before fixation for immunofluorescence.

### *De novo* transformation

pLenti-PGK-KRAS4B^G12C^ was generated by PCR-based mutagenesis of an available pLenti-PGK-KRAS4B^G12V^ plasmid (Addgene, #127233). Briefly, *KRAS* was PCR amplified from the vector into two fragments inserting the desired mutation by amplifying the mutagenized region with primers listed in Supplementary Table S5. The two PCR fragments were gel purified and used as template for a second-stage PCR were the full length *KRAS*^G12C^ was generated using the external primers from the previous PCRs. Vector and *KRAS*^G12C^ complementary DNA (cDNA) were then digested and ligated and mutagenesis was verified by Sanger sequencing. HCEC-1CT cells were transfected with gRNA targeting *TP53* as described above, and the polyclonal *TP53* knockout population was expanded for 1 week. Lentiviral particles containing pLenti-PGK-KRAS4B^G12C^ were generated as described above, and the *TP53* knockout population was infected. After 1 week of further expansion, cells were plated for soft agar assay, quantitative reverse transcription polymerase chain reaction (RT-qPCR) detection of exogenous *KRAS*, and immunofluorescence validation of TP53 depletion.

### Soft agar assay

To prepare soft agar in six-well plates, a bottom layer of 1.5 ml 0.5% seaplaque agarose (Lonza, 50 100) in MEM media with supplements was poured and cooled, and 5000 cells suspended in 1.5 ml 0.4% Seaplaque soft agar in supplemented MEM was poured on top. Cells were incubated for 10–14 days under layer of fresh cell culture medium which was changed every 2 days. For analysis, 500 μl of 100 μg/ml thiazolyl blue tetrazolium bromide (Merck, M2128) was added in each well overnight and plates scanned at 1200 dpi on an Epson Perfection V700. Images were analysed using ImageJ version 2.1.051.

### Mass-spectrometry profiling of histone post-translational modifications

Cells were cultured with or without 50 nM Quisinostat (Insight Biotechnology, HY-15433-1ml) for 16 h before histone samples were prepared for mass-spectrometry (MS) analysis. Histones were enriched from 1 × 10^6^ cells as previously described [45]. Approximately 4 μg of histone octamer were mixed with an equal amount of heavy-isotope labelled histones, which were used as an internal standard (super-SILAC mix) [46] and separated on a 17% sodium dodecyl sulfate–polyacrylamide gel electrophoresis gel. Histone bands were excised, chemically acylated with propionic anhydride and in-gel digested with trypsin, followed by peptide N-terminal derivatization with phenyl isocyanate [47]. Peptide mixtures were separated by reversed-phase chromatography on an EASY-Spray column (ThermoFisher Scientific), 25-cm long (inner diameter 75 μm, PepMap C18, 2-μm particles), which was connected online to a Q Exactive Plus instrument (ThermoFisher Scientific) through an EASY-Spray™ Ion Source (ThermoFisher Scientific), as described [47]. The acquired RAW data were analyzed using EpiProfile 2.0 [48], selecting the SILAC option, followed by manual validation. For each histone-modified peptide, a % relative abundance (%RA) value for the sample (light channel – L) or the internal standard (heavy channel – H) was estimated by dividing the area under the curve of each modified peptide for the sum of the areas corresponding to all the observed forms of that peptide and multiplying by 100. Light/heavy (L/H) of %RA were then calculated and are reported in Supplementary Table S3. The MS data have been deposited to the ProteomeXchange Consortium [49] via the PRIDE partner repository with the dataset identifier PXD039819.

### Chromatin immunoprecipitation followed by sequencing (ChIP-seq)

Parental and monoclonal *CREBBP*-KO human colonic epithelial cell (HCEC) cells were cross-linked by addition of formaldehyde to a final concentration of 1%, and incubation for 10 min at room temperature. Fixation was stopped by addition of glycine to a final concentration of 0.125 M. Cells were washed in phosphate-buffered saline, harvested in sodium dodecyl sulfate (SDS) buffer [50 mM Tris (pH 8.1), 0.5% SDS, 100 mM NaCl, 5 mM ethylenediaminetetraacetic acid (EDTA), protease inhibitors], spun down, and resuspended in ice-cold IP buffer [1 vol. SDS buffer, 0.5 vol. Triton dilution buffer {100 mM Tris–HCl (ph 8.6), 100 mM NaCl, 5 mM EDTA, 5% Triton X-100}, protease inhibitors]. Samples were then sonicated to an average length of 500–200 bp, using a Bioruptor Pico sonifier at 30% amplitude. The volume of the samples was adjusted with IP buffer to reach ∼1 ml per immunoprecipitation (9 μg of DNA per sample). The sheared chromatin was precleared with blocked Protein-A beads (Cytiva, #GEH17078001). After removing 5% of the lysate to be used as the input samples, the lysates were incubated with 4 μg anti-H3K27ac antibodies (Abcam, Ab4729), followed by overnight incubation at 4°C on a rotating wheel. The next day, lysates were centrifuged for 20 min at full speed, and the supernatants were transferred to clean Eppendorf tubes pre-loaded with blocked protein A beads, followed by incubation for 3 h at 4°C on a rotating wheel. The beads were then centrifuged and washed three times in Mixed Micelle Washing Buffer [150 mM NaCl, 20 mM Tris–HCl (pH 8.1), 5 mM EDTA, 5.2% (w/v) sucrose, 1% Triton X-100, 0.2% SDS], three times in Buffer 500 (0.1% deoxycholic acid, 1 mM EDTA, 50 mM HEPES, 500 mM NaCl, 1% Triton X-100), three times in LiCl/detergent solution buffer [0.5% deoxycholic acid, 1 mM EDTA, 250 mM LiCl, 0.5% NP-40, 10 mM Tris–HCl (pH 8)], and once more in Tris-EDTA (TE) buffer, before final resuspension in TE with 2% SDS and overnight incubation at 65°C for reversing the cross-links. Finally, the beads were discarded by centrifugation and the supernatants moved to clean Eppendorf tubes, followed by DNA purification using a Qiaquick PCR Purification kit (Qiagen, 28106). Sequencing libraries were prepared using the NEBNext Ultra II DNA Library Prep Kit 96 (New England Biolabs, BE7645L) according to manufacturer’s instructions. Adapter ligation was carried out using the NEBNext Multiplex Oligos for Illumina (96 Dual Index Pairs) according to manufacturer’s instructions. The quality and fragment size distributions of the purified libraries was assessed by a 4200 TapeStation Instrument (Agilent Technologies). Libraries were sequenced on a NovaSeq 6000 (Illumina) to an average read depth of 30M.

### ChIP-seq analysis

#### Alignment

FASTQ files for each sample were processed via the ChIP-seq pipeline v2.0.0 available at nf-core [50] against the *Homo sapiens* hg38 genome with narrow peak calling with MACS2. Generated Bam and BigWig files were used for inspection. To analyze samples with a comparable sequencing depth, FASTQ files were downsampled to a maximum of 36M reads per sample using seqtk (version 1.4-r122). Generated FASTQ files were then processed via nf-core as described above.

#### Peak calling

Consensus peak matrix generated through MACS2 was used as input. Peaks were filtered by fold change (equal or greater than 5) and presence in all the three replicates of the same condition.

#### Differential peak calling

Differential peak analysis was performed with the DiffBind package [51] within the R programming environment (version 4.4.0). The analysis was performed on the peak calling from MACS2, with default parameters. Only peaks with a log_2_ concentration of 5 in at least one condition were retained. Peaks were defined as differential if meeting the following criteria: log_2_ fold change ≥ 1, false discovery rate (FDR) < 0.0001.

### RNA-seq

Total RNA was isolated using a RNeasy Plus Mini Kit (Qiagen, 74136). Sequencing libraries were prepared using the KAPA mRNA HyperPrep Kit (Roche, KK8581) according to manufacturer’s instructions. Adapter ligation was carried out using the KAPA Unique Dual-Indexed Adapters Kit (15 μM) (Roche, KK8727) according to manufacturer’s instructions. To remove short fragments such as adapter dimers, two SPRISelect bead clean-ups were done (0.63× SPRI and 0.7× SPRI). The library was amplified with KAPA HiFi HotStart PCR Master Mix plus Library Amplification Primer Mix in 13 PCR cycles as recommended by the manufacturer. Amplified libraries were purified via a SPRISelect 1× bead cleanup. The quality and fragment size distributions of the purified libraries was assessed by a 4200 TapeStation Instrument (Agilent Technologies). Libraries were pooled and sequenced on the HiSeq4000 (Illumina) in SR100 configuration to an average read depth of 25M. Adapter trimming was performed with cutadapt (version 1.9.1) [52] with parameters “–minimum-length = 25 –quality-cutoff = 20 -a AGATCGGAAGAGC”. The RSEM package (version 1.3.0) [53] in conjunction with the STAR alignment algorithm (version 2.5.2a) [54] was used for the mapping and subsequent gene-level counting of the sequenced reads with respect to hg19 RefSeq genes downloaded from the UCSC Table Browser [55]. The parameters used were “–star-output-genome-bam –forward-prob 0”. Transcript per million (TPM) values were calculated to quantify expression levels of ERGs.

### RT-qPCR

RNA was isolated from three independent samples using RNeasy Plus Mini Kit (Qiagen, 74 136) according to the manufacturer’s instructions. A total of 500 ng of RNA was reverse transcribed using a High Capacity cDNA Reverse Transcription Kit (ThermoFisher, 4368814) according to the manufacturer’s instructions. cDNA was diluted 1 in 10 and used as input for RT–qPCR using Advanced Universal SYBR Green Supermix (Bio-Rad, 172-5274) on a CFX96 real-time PCR detection system (Bio-Rad) using primers listed in Supplementary Table S5.

### Public data analysis

Fitness values for ERG-KO cancer cell lines were obtained from the DepMap project by accessing available Chronos scores for 1150 cell lines in release 24Q2 [56]. Interaction networks for each gene of interest were accessed from the STRING database version 11.59 (https://string-db.org/). The full STRING network and physical subnetwork were taken for all highest confidence interactors (combined interaction score > 0.9). Network graphs were visualized and analyzed in Cytoscape 3.9.0. Layouts were plotted using an edge-weighted spring-embedded layout using the STRING combined interaction score. Paralogue homology was retrieved the ClustalW (1.81) alignment from Ensembl (release 109). Driver information for ARID1A and CREBBP were accessed from Network of Cancer Genes version 7.0 (http://ncg.kcl.ac.uk/). Cancer-associated mutations were accessed from the curated set of nonredundant pan-cancer studies from cBioPortal v5.2.0 (https://www.cbioportal.org/). Inhibitor sensitivity data was retrieved from GDSC2 (https://www.cancerrxgene.org/) [57].

## Results

### Most epigenetic regulators are dispensable for the maintenance of human somatic cell fitness

**Figure 1.**
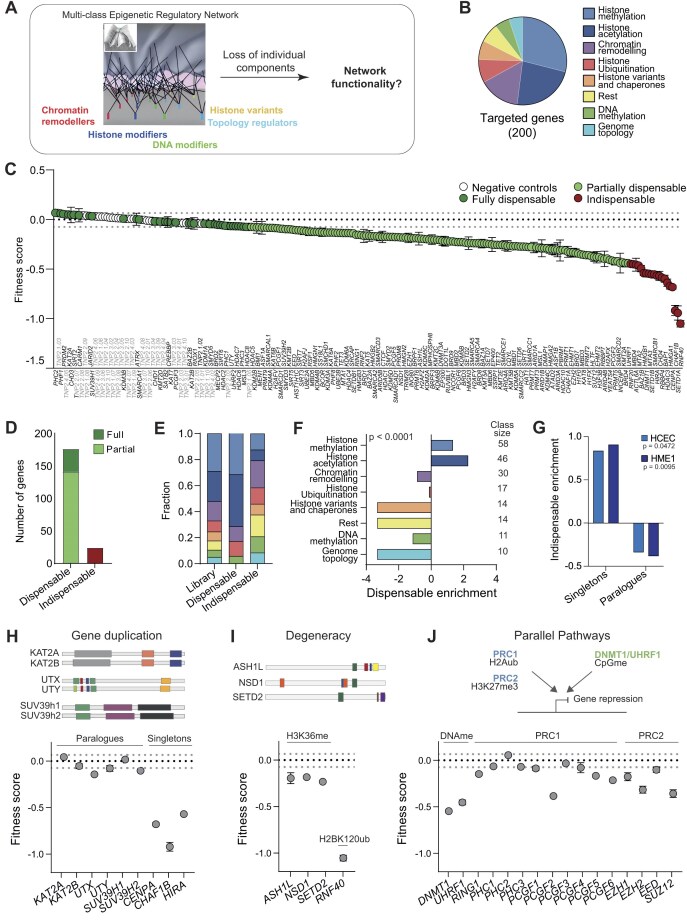
Normal human epithelial cells are robust to epigenetic perturbation. (**A**) Schematic of the experimental approach. The bottom view of the Waddington landscape is used to visualize functional connections within and across ERN functional classes that sustain the cell epigenetic landscape. Image adapted from Rajagopal and Stanger [83] with permission from Elsevier. (**B**) Proportion of targeted genes in each functional group. (**C**) Profile of fitness scores in HCECs. Values: mean ± standard error of the mean (SEM) of three replicates. Dotted lines: mean and minimum/maximum negative control values, respectively. (**D**) Classification of ERGs in HCECs based on results in panel (C). Fraction (**E**) and enrichment (**F**) of fully dispensable or indispensable ERGs across functional class. Legend for ERN classes as in panel (B). Values in F are the log_2_ ratio of observed versus expected dispensable proportion. Statistical significance of the enrichment (two-tailed χ^2^ test, 7 df) and the size of each functional class are indicated. (**G**) Enrichment of genes with or without paralogues amongst indispensable ERGs, as the log_2_ ratio of observed versus expected counts. Paralogues for ERGs retrieved from Ensembl release 93 with sequence identity ≥ 0.2 [84]; histone variants were manually included. Statistical significance of the enrichment in each line (two-tailed χ^2^ test) is indicated. (**H**–**J**) Fitness scores for HCEC populations with the indicated genes knocked-out. The structure of the corresponding proteins retrieved from cBioPortal is indicated in panel (H) and (I), while a schematic diagram illustrates parallel pathways mediating gene silencing in J.

To uncover principles underpinning the robustness of the ERN in human somatic cells, we employed a genetic approach to systematically perturb the network and define the extent of disruption that leads to its collapse (Fig. 1A).

A challenge in dissecting functional interactions in the ERN is that perturbation of epigenetic regulators impacts various aspects of genome function, including gene transcription, DNA replication, repair of genotoxic lesions, and packaging of DNA within the nucleus, which makes it difficult for a single molecular readout to capture all interactions. Inspired by epistasis studies in yeast, we opted to use cell fitness as an integrated phenotype that reflects the efficient and coordinated execution of all these nuclear processes, and uncovers genetic interactions without a priori knowledge of the affected pathways. While fitness readouts do not capture subtle molecular changes, or alterations impacting organismal function (e.g. developmental or aging-related defects), perturbations that have detectable cellular consequences in homeostatic conditions uncover fundamental frameworks that sustain ERN integrity and reveal the relative contribution of individual network components.

To interrogate ERN robustness in a physiological setting, we selected as an initial model system human colonic epithelial cells. HCECs are karyotypically normal cells isolated from a healthy adult individual (Supplementary Fig. S1A), which retain expression of intestinal markers and physiological properties such as the ability to form morphologically normal organoid cultures when grown in Matrigel [58]. Expression of the cyclin-dependent kinase 4 and human telomerase reverse transcriptase (hTERT) allow HCECs to avoid replicative senescence [58], making them a suitable system to study how experimental perturbation impacts cell fitness in the absence of confounding effects. Moreover, the high cellular turnover of the normal intestinal epithelium makes cell fitness a physiologically relevant feature in the context of tissue homeostasis. To be able to temporally control gene inactivation, we transduced HCECs with doxycycline-inducible Cas9 (iCas9) [39] and isolated a clone that enables efficient induction of CRISPR-induced in/dels (80–90% gene editing). Due to random in-frame in/dels that lead to KO escape, ∼70% of cells within the population typically lacked the targeted proteins 3 days after Cas9 induction (Supplementary Fig. S1B). Using this experimental system and a focused lentiviral sgRNA library [42], we systematically inactivated 200 epigenetic regulators across eight functional classes and examined the effect of this perturbation on cellular fitness over the course of 11 days (Fig. 1B, Supplementary Fig. S1D, and Supplementary Table S1).

As a positive control, inactivation of the essential ribosomal gene *RPL12* strongly impacted cell fitness, with cell count reduced by >95% in the KO population after 11 days (Supplementary Fig. S1C). We generated knockout populations lacking individual ERGs in 96-well format and assessed their fitness relative to control populations where the nonexpressed testis-specific *TNP2* gene was targeted (10 control populations in each plate, see the “Materials and methods” section) (Supplementary Fig. S1D). The distribution of fitness scores (log_2_-transformed cell count for a given gene normalized to the average of negative control values on the plate) showed a continuum of effects, with most populations exhibiting fitness comparable to the negative controls, or mildly decreased, and only a minority of them deviating from the overall trend (Fig. 1C and Supplementary Table S2). Genes indispensable for network functionality, defined as those whose inactivation resulted in a fitness score lower the half-maximal effect, were 12% of the targeted genes (Fig. 1C and D). Higher fitness scores measured for the other 176 knockout populations indicated that the corresponding gene products exert dispensable functions, either entirely (fitness scores within the observed range of negative control values) or partly (Fig. 1C and D). The fitness profile was not determined by varying efficiency of gene editing, as sgRNAs targeting indispensable or dispensable genes induce indels with comparable efficiency [42] (*P* = .36 one-way ANOVA, *P* = .53 Pearson correlation) (Supplementary Fig. S1E). Fully dispensable and indispensable genes were differentially distributed across functional classes (*P* < .0001, two-tailed χ^2^ test), with fully dispensable genes being enriched for histone modifiers, especially those involved in lysine acetylation and methylation, and depleted of histone variants, their chaperones, and regulators of genome topology (Fig. 1E and F). Interestingly, large functional classes comprising >40 components were depleted of indispensable genes, in line with a high degree of functional redundancy among those components, whereas no fully dispensable genes were observed within smaller classes (Fig. 1F). Moreover, indispensable genes were depleted of paralogues evolved via gene duplication (Fig. 1G), as exemplified by genes encoding the histone acetyl transferases KAT2A/B, the histone methyltransferase SUV39H1/2, and the histone demethylases KDM6A/C (UTX/UTY) (Fig. 1H). Among dispensable ERN components we also observed several histone modifiers known to share common substrates; for instance, ASH1L, NSD1, and SETD2 are structurally unrelated enzymes that belong to distinct protein complexes, but all methylate H3K36 via degenerate pathways [24], and their knockout had minimal effect on HCECs fitness (Fig. 1I). In contrast, inactivation of RNF40, the catalytic subunit of the BRE1 complex uniquely responsible for ubiquitination of H2BK120 [59], was strongly deleterious (Fig. 1I). In agreement with the fitness phenotype, levels of histone mark deposited by redundant chromatin modifiers were not altered upon KO of the respective genes (Supplementary Fig. S1F and G), whereas inactivation of RNF40 led to substantial reduction of the cognate modification (Supplementary Fig. S1G and H). These observations link cellular and molecular effects of ERN disruption and provide evidence that cellular fitness is a good proxy for network robustness. We also examined an additional layer of redundancy, focusing on parallel pathways that regulate gene silencing through independent mechanisms: DNA methylation and facultative heterochromatin. Disruption of the polycomb repressive complexes had mild to moderate effects, while inactivation of the maintenance DNA methyltransferase DNMT1 and its targeting factor UHFR1 were more deleterious (Fig. 1J). The observed pattern suggests that the two classes of modifiers exert nonoverlapping functions in repressing transcription and/or genomic instability, but neither of them is indispensable for network integrity. Overall, the initial analysis using HCECs indicates that the loss of most epigenetic regulators is tolerated by normal human cells, and that only a few network components, mainly exerting structural functions, are essential to sustain somatic cell fitness.

To assess the generality of the observed patterns, we extended the analysis to normal cells of a distinct lineage: hTERT-immortalized, mammary epithelial HME1 cells, isolated from a mammary gland of a healthy individual. Using the same experimental strategy employed for HCECs, we isolated a clone of iCas9-expressing HME1 cells characterized by high KO efficiency and performed the large-scale fitness assay (Supplementary Fig. S1B–D). The general trend was similar to that observed with HCECs, with 83% of targeted genes classified as dispensable (Supplementary Fig. S2A and B and Supplementary Table S2), 95% of which overlapped with dispensable genes identified in HCECs (Supplementary Fig. S2E). As observed in HCECs, histone modifiers were also enriched among dispensable genes in HME1s, while the indispensable gene set contained several genome topology regulators (Supplementary Fig. S2C and D) and was enriched for singletons (Fig. 1G). Individual fitness scores from the two cellular models were highly correlated (*P* < .0001, Pearson correlation), and genes associated with the most deleterious effects were clearly separated from the bulk of the dataset, confirming that unique properties characterize this small subset of epigenetic regulators, while core cell functionality is preserved in the absence of most ERGs (Supplementary Fig. S2F). Specific patterns were also independent of the tissue-of-origin, as exemplified by degenerate enzymes cooperating in H3K36me3 deposition or parallel pathways maintaining gene silencing (Supplementary Fig. S2G).

Comparing fitness effects observed in noncancerous HCEC and HME1 with data from the Cancer Dependency Map Project (DepMap) [13] confirmed the overall robustness of the ERN, but also highlighted the need of interrogating the network in the absence of disease-induced confounding effects (Supplementary Fig. S3). While the median value of fitness scores across 1150 cancer cell lines mirrored the profiles observed in HCEC and HME1 cells, with only a minority of ERGs deviating from the overall trend (Supplementary Fig. S3A and B), individual values varied substantially, and several genes defined as essential (Chronos score < −1) in some lines were dispensable in others (Supplementary Fig. S3A and C). Notably, even ERGs whose inactivation was consistently deleterious, showed milder effects than essential genes such as RPL17 (Supplementary Fig. S3C).

We conclude that robustness is an intrinsic property of the ERN in human somatic cells, supporting cellular fitness across diverse tissue types in disease-free settings. The loss of individual components is overall well-tolerated and only a small subset of genes is essential for core cell functionality. For other genes, a combination of duplicated components and degenerate pathways within and across functional classes appears to buffer the effects of perturbation.

### Resilience of the ERN to the combined loss of paralogue pairs

**Figure 2.**
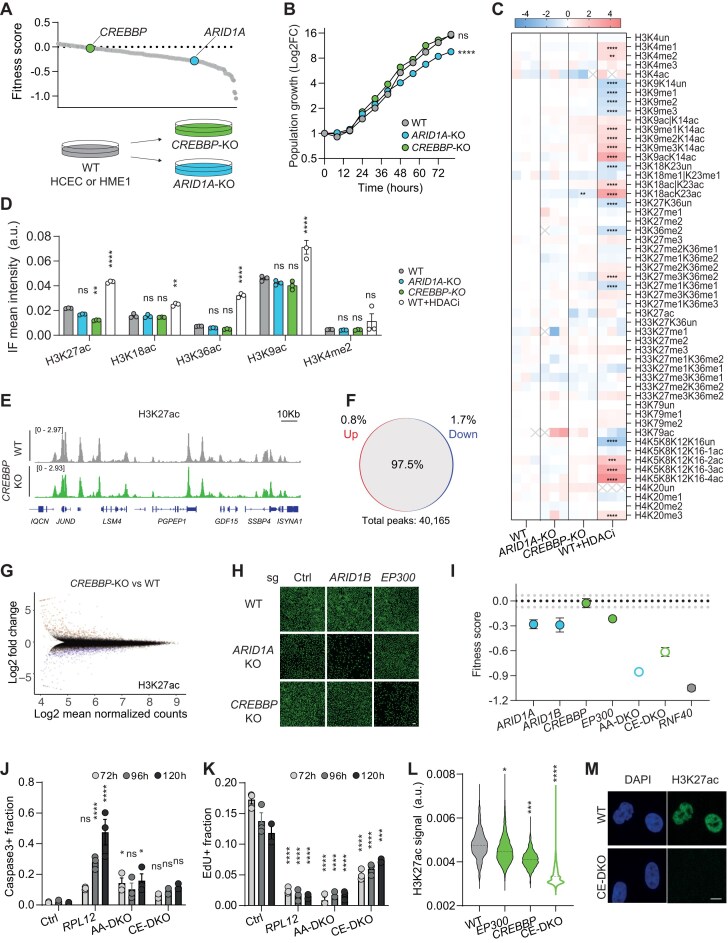
Loss of paralogue pairs does not induce ERN collapse. (**A**) Upper, *ARID1A* and *CREBBP* highlighted on the profile of HCEC fitness scores. Lower, schematic of knockout line generation. (**B**) Proliferation curve of the indicated HCEC isogenic lines. Values: mean ± SEM of six replicates. *T*-test comparing the slope of a linear model fitted to the log_2_-transformed values between 24 and 48 h, four asterisks: two-tailed *P* < .0001. (**C**) Log_2_ of L/H ratio (where L = sample and H = internal standard) relative to the mean value of WT cells for the indicated differentially modified histone peptides in the isogenic HCEC lines, and WT cells treated with 50 nM Quisinostat (HDACi). Three biological replicates per condition are shown. One, two, three and four asterisks: *P* < .05, *P* < .01, *P* < .001, and *P* < .0001, respectively. Cross: undetected peptides. (**D**) Quantitative immunofluorescence microscopy of single cells from the indicated populations. Median values of the signal intensity distributions from three biological replicates are plotted. Two, and four asterisks: *P* < .05 and *P* < .0001, respectively. a.u.: arbitrary units. (**E**) ChIP-seq tracks of H3K27ac in the indicated lines showing unaffected patterns. (**F**, **G**) Quantification of differential H3K27ac peaks detected in *CREBBP*-KO cells visualized as venn diagrams (F) or MA-plot (G). Visualization (H) and quantification (I) of fitness impact by the indicated sgRNAs in the isogenic HCEC lines. Nuclei were stained with SYTOX green. Ctrl: control, *TNP2*-targeting sgRNA. Values: mean ± SEM of three biological replicates. Black and grey dotted lines: mean and minimum/maximum negative control values, respectively. Fraction of apoptotic (J) and proliferating cells in S-phase (K) at the indicated time after knockout of the indicated genes. Values: mean ± SEM of three biological replicates. Two-way ANOVA with Dunnett’s multiple comparison test, samples compared at each timepoint against Ctrl. One, two, three and four asterisks: *P* < .05, *P* < .01, *P* < .001, and *P* < .0001, respectively. (**L**-**M**) Quantitative immunofluorescence microscopy of HCECs upon single or double inactivation of *CREBBP* and *EP300*. Distributions of H3K27ac signal intensity in single cells (L) and representative images (M). n = 431–2571, two-tailed t-test of three independent population medians versus WT; one, three, and four asterisks: *P* < .05, *P* < .001, and *P* < .0001, respectively. Scale bar: 10 μm.

To directly examine how functional interactions among epigenetic regulators preserve network integrity upon loss of one component, we focused on two genes whose inactivation is either fully (*CREBBP*) or partly (*ARID1A*) tolerated in both HME1 and HCEC cells (Fig. 2A).

CREBBP, also known as CBP or KAT3A, is a histone modifier that acetylates multiple lysine residues on histone H3 and H4 and plays a key role in enhancer activation [60, 61]. ARID1A is a member of the ATP-dependent chromatin remodeling complex SWI/SNF, which regulates nucleosome positioning [62]. ARID1A has no enzymatic activity and is thought to recruit the cBAF complex to its target sites through direct binding to DNA and protein–protein interactions [63]. Although their molecular functions are distinct, both CREBBP and ARID1A mainly act as transcriptional co-activators, and they are also involved in DNA replication and genome maintenance [26, 64]. Both genes are ubiquitously expressed, have regulatory interactions with other genes and noncoding RNAs, and the corresponding products physically interact with many other proteins (Supplementary Fig. S4A and B). Highlighting their functional importance, genetic alterations in *ARID1A* and *CREBBP* often have pathological consequence: germline substitutions lead to developmental disorders, including Coffin-Siris syndrome 2 (*ARID1A* loss-of-function mutations), Rubinstein–Taybi syndrome (*CREBBP* loss-of-function mutations) and Menke–Hennekam Syndrome (*CREBBP* missense mutations) [32], while somatic alterations are recurrently observed across multiple cancer types (Supplementary Fig. S4C and D).

We isolated clonal HCEC and HME1 lines lacking either ARID1A or CREBBP (Fig. 2A and Supplementary Fig. S4E–H). In agreement with the results of the large-scale assay, the *CREBBP*-KO population exhibited unaltered growth kinetics compared with wild-type (WT) cells, whereas *ARID1A*-KO cells proliferated at a slower rate (Fig. 2B and Supplementary Fig. S2H). Even though inactivation of CREBBP had no detectable impact on cell fitness, we asked whether molecular features of chromatin responded to its loss. To this end, we profiled a panel of histone modifications in the three isogenic lines by quantitative MS, an accurate method that quantifies relative levels of up to 60 unmodified or modified histone peptides [65]. While this analysis is particularly relevant for *CREBBP*-KO cells, we reasoned that indirect changes could be induced in *ARID1A*-KO cells as a consequence of altered transcription or genome maintenance. As a positive control, we included in the analysis WT cells treated with Quisinostat, a broad-spectrum HDAC inhibitor (HDACi). As expected, we observed differential abundance (-0.41 < log_2_FC < 0.58 corresponding to a fold decrease or increase >1.5,*P*-value < .01) of multiple histone marks in HDACi-treated HCECs, with 12 and 9 peptides showing increased and decreased levels, respectively (Fig. 2C). In contrast, histone marks were largely unaffected in both *ARID1A*-KO and *CREBBP*-KO cells, with differential levels only detected for the H3K18acK23ac co-modified peptide in *CREBBP*-KO cells (39% reduction, *P* = .0016) (Fig. 2C and Supplementary Table S3). H3K27ac exhibited a moderate reduction in CREBBP-KO cells, of border-line statistical significance (53% reduction, *P* = .07) (Fig. 2C). Similar patterns were also observed in HME1 cells, with no peptide showing differential levels in KO cells, including direct CREBBP substrates (Supplementary Fig. S2I and Supplementary Table S3). To validate these results using an orthogonal method, and to extend the analysis to other modifications that could not be detected by MS, we performed quantitative immunofluorescence in HCECs using antibodies specific to H3K27ac, H3K18ac, H3K36ac (established CREBBP-deposited marks), H3K9ac (a CREBBP-independent acetylation mark), and H3K4me2 (a mark showing unaltered levels in KO cells by MS). In agreement with the MS analysis, all acetylation marks strongly increased upon HDACi treatment, but were not substantially affected in the KO populations: H3K27ac was significantly decreased in *CREBBP*-KO cells, but the effect size was minimal compared with the change induced by HDACi (Fig. 2D). To examine potential redistribution of the mark across the genome, we performed ChIP-seq analysis: 97.5% of H3K27ac peaks were unaffected by the loss of CREBBP, with the few differences mainly observed in low-magnitude peaks (Fig. 2E–G). While changes in other epigenetic features in the KO cells cannot be excluded, the observed patterns indicate that their chromatin landscape is largely unaltered. These observations confirm that both regulators are dispensable for overall ERN functionality in steady-state conditions, and suggests that, in their absence, compensatory mechanisms maintain homeostatic levels of many modifications linked to transcription and genome maintenance.

Primary candidates for functional compensation in ARID1A- and CREBBP-deficient cells are the two respective paralogs ARID1B and EP300, also known as p300 or KAT3B. Both pairs of paralogs have evolved through duplication of a common ancestor gene and are characterized by highly homologous protein domains. ARID1A and ARID1B share ∼80% of amino-acid sequence, whereas the CREBBP-EP300 pair displays a lower sequence homology of ∼63%. Indeed, acute inactivation of the respective paralogue had deleterious effects in *ARID1A*-KO or *CREBBP*-KO cells, with *ARID1A/B* double-KO cells (AA-DKO) showing a stronger fitness reduction than the *CREBBP-EP300* double-KO population (CE-DKO; Fig. 2H and I). Interestingly, the combined loss of each pair was not as detrimental as the inactivation of individual indispensable genes such as *RNF40*, suggesting that other compensatory mechanisms sustained partial network functionality in DKO cells (Fig. 2I). The reduced fitness of DKO cells was mainly due to inhibited proliferation, as assessed by EdU incorporation and caspase 3 activation assays, with a significant drop in the fraction of cells in S-phase and only a minor increase in the percentage of apoptotic cells, unlike *RPL12*-deficient cells (Fig. 2J and K). In line with a relatively higher fitness score, CE-DKO cells also displayed a milder reduction in proliferating cells (∼2-fold lower than control cells) (Fig. 2K), further indicating that even in the absence of two major histone modifiers playing key roles in transcriptional activation, the network was only partly affected and did not collapse. Of note, the effect of CREBBP/EP300 combined loss was readily detectable at the molecular level, with H3K27ac levels dropping to background ones in CE-DKO cells (Fig. 2L and M). Altogether, these observations support the notion that gene duplication is an important mechanism that contributes to ERN robustness, but they also highlight interesting differences in the degree of compensation provided by distinct paralogues, as well as differential resilience of the ERN to the combined loss of paralogue pairs: loss of ARID1A in the presence of ARID1B is more deleterious than loss of CREBBP in the presence of EP300, and AA-DKO are more severely disrupted than CE-DKO.

### Functional interactions within and across classes of epigenetic regulators

**Figure 3.**
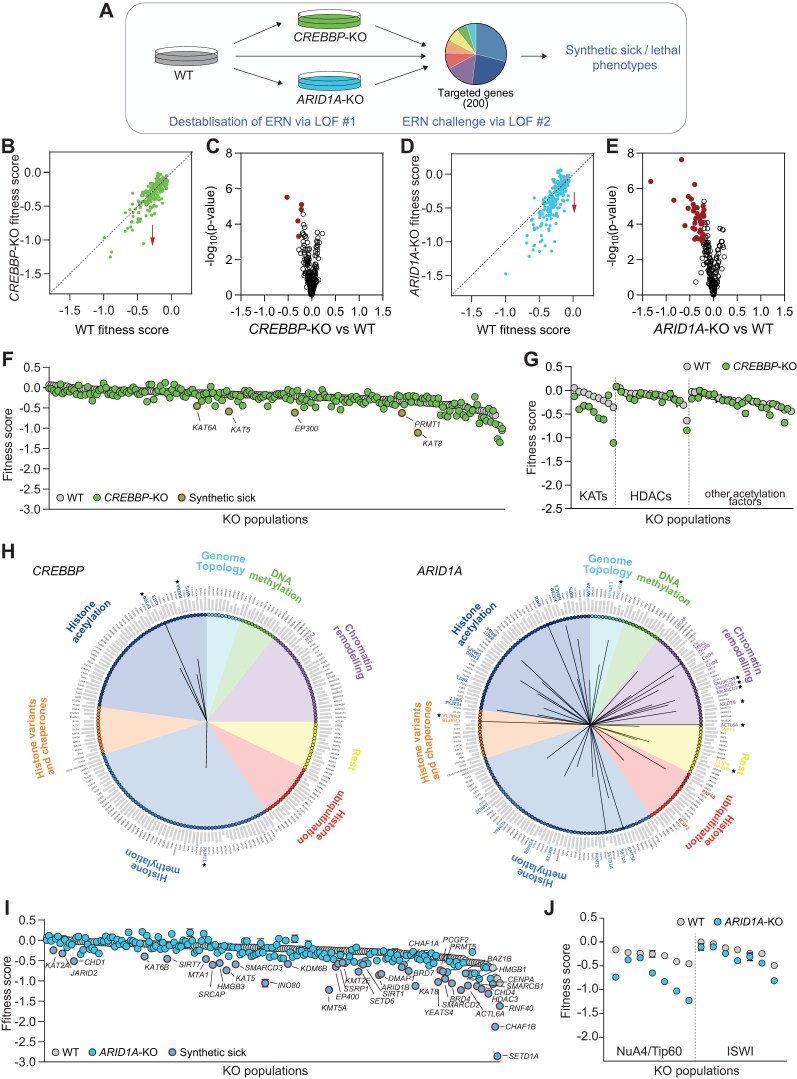
Functional interactions of ARID1A and CREBBP within the ERN. (**A**) Schematic of the experimental approach used to identify functional interactions that sustain ERN functionality in or *CREBBP- or ARID1A*-KO cells. Fitness scores of ERG-KO populations in WT and *CREBBP*-KO (**B**) or *ARIDA1*-KO (**D**) HCEC cells. Values: mean ± SEM of three biological replicates. Vertical drop from line of identity (red arrow) indicates synthetic sick phenotypes. (**C** and **E**) Identification of functional interactions based on linear regression analysis. *x*-axis: regression coefficient, *y*-axis: Wald-test p-value. Red symbols: interactors whose loss synergizes with CREBBP (C) or ARID1A (E) loss. Fitness scores of ERG-KO Populations in WT and *CREBBP*-KO (F) or *ARID1A*-KO (I) HCEC cells. Values, mean ± SEM of three biological replicates. Red outline symbols: DKO populations exhibiting synthetic sick phenotypes based on linear regression analysis. (**G**, **J**) Fitness score of the indicated KO populations in WT and *CREBBP*-KO (G) or *ARID1A*-KO (J) HCEC cells. (**H**) Identified CREBBP or ARID1A functional interactions with ERN components in HCEC cells arranged by functional class. Edges are scaled to indicate interaction effect size, up to a maximum absolute coefficient value of 1. Gray bar, fitness score in WT cells. Black star: physical interactor by STRING database.

To comprehensively identify intra- and inter-class functional interactions that sustain ERN functionality, we repeated the large-scale fitness assay in *CREBBP*-KO and *ARID1A*-KO cells, searching for DKO populations that exhibited a synthetic lethal/sick phenotype, i.e. a significantly decreased fitness relative to the single KO population generated from parental, WT cells (Fig. 3A). Importantly, the assay was performed in parallel in *CREBBP*-KO and *ARID1A*-KO cells, so that the two genotypes would control for each other and results could be directly comparable, minimizing technical variability. DKO cells in the *CREBBP*-KO background displayed largely unaffected fitness, with fitness scores of single and double KO populations lying on the diagonal line of identity in most cases (Fig. 3B, genetic interactions revealed by a vertical drop, as indicated by the red arrow). Fitness profiles of single- and double-KO cells were highly similar, and through regression analysis based on the distribution of multiple negative controls, we identified only five populations showing synthetic-sick phenotypes (*P* < 1 × 10^−3^, regression coefficient > 0.20) (Fig. 3C and F). In addition to highlighting the consistency of our assay across experiments, this result indicates that the ERN can effectively buffer the combined loss of CREBBP and most ERGs (Fig. 3F). Strikingly, out of the five genetic interactors identified, four were other histone acetyltransferases (KATs) (Fig. 3F and H and Supplementary Tables S2 and S4). Inspection of selected DKO populations revealed that most targeted KATs showed a functional interaction with CREBBP while inactivation of other acetylation regulators had no effect (Fig. 3G). These observations suggest that intra-class functional compensation may sustain ERN integrity in the absence of CREBBP or other KATs, and only when cells lack more than one enzyme, robustness begins to be compromised. Notably, KAT5- or KAT8-deficient DKO cells displayed comparable or stronger phenotypes than DKO cells lacking EP300, suggesting that functional compensation via degeneracy may be as important as that provided by partly divergent paralogues (Fig. 3F). HME1 cells lacking CREBBP exhibited similar patterns as HCECs, with broadly unaffected fitness of DKO cells and enrichment of KATs among the few functional interactors (Supplementary Fig. S5A and B).

We then asked whether *ARID1A*-KO cells respond differently to the loss of a second epigenetic regulator. In contrast to CREBBP-KO cells, HCECs lacking ARID1A displayed overall compromised ERN functionality, as indicated by a broadly decreased fitness of DKO populations relatively to the corresponding single KO ones (Fig. 3D, vertical drop from the diagonal line of identity). Despite the widespread effect, most DKO populations exhibited synthetic sick phenotypes of moderate magnitude, indicating that ERN functionality was only partly affected (Fig. 3E and I and Supplementary Table S2). We identified 38 functional interactors of ARID1A (Fig. 3E and H and Supplementary Table S4), and validation experiments on genes representing the full range of detected phenotypes confirmed the accuracy of the large-scale analysis (Supplementary Fig. S5C). Notably, synthetic sick phenotypes spanned across the entire ERN, with no epigenetic regulator class either enriched or depleted (Fig. 3H). As expected, ARID1A functional interactors included other subunits of the SWI/SNF complex (Fig. 3H and Supplementary Fig. S5D) and additional proteins that have been shown to physically interact with ARID1A (Fig. 3H black star-labelled interactors). However, 78.9% of the observed genetic interactors have not been reported to bind to ARID1A (Fig. 3H interactors without star), pointing to parallel pathways — rather than protein–protein interactions — as a prominent mechanism that supports ERN integrity in the context of ARID1A. In line with the notion that SWI/SNF and the ISWI complex exert distinct functions in remodeling chromatin [66], we did not detect substantial interactions between ARID1A and ISWI subunits (Fig. 3J). Instead, members of the NuA4/Tip60 acetyltransferase complex were amongst the strongest interactors, suggesting potential synergistic functions in the context of genome maintenance or transcriptional regulation [67, 68] (Fig. 3J). Confirming the patterns detected in HCECs, ARID1A-deficient HME1s also exhibited broadly compromised ERN robustness and network-wide functional interactions, independent of physical binding (Supplementary Fig. S5A and B). These results suggest that ARID1A acts as a key functional hub within the ERN in multiple lineages, and show that its absence sensitizes cells to the combined loss of many other epigenetic regulators. Although interactions revealed through combinatorial mutagenesis were numerous, the ERN was remarkably resilient even upon loss of two epigenetic regulators, and the net effect on cell fitness was mild in most cases (Fig. 3I).

We conclude that multiple modalities have evolved to build resilience in the ERN: (i) hubs such as ARID1A functionally interact with many proteins across all classes and its loss broadly destabilizes the network; (ii) the absence of other key regulators such as CREBBP is buffered via intra-class compensation, with multiple KATs, characterized by either common or distinct substrate specificity, exerting synergistic functions.

### Synthetic fragility of the ERN upon transformation

**Figure 4.**
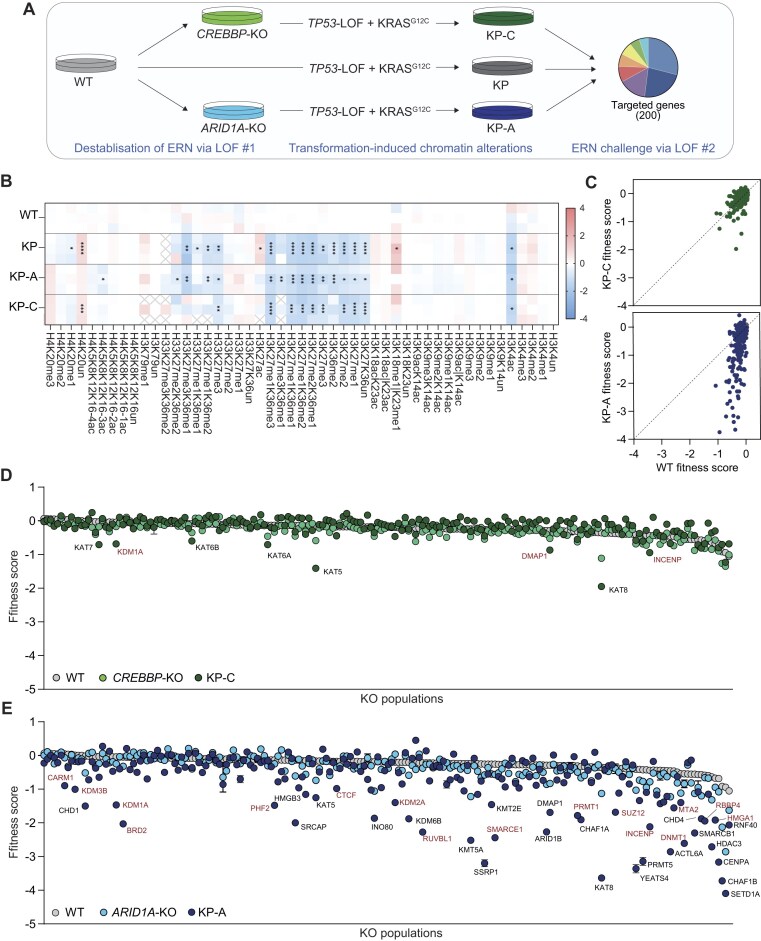
Synthetic fragility of the ERN upon transformation. (**A**) Schematic of the experimental approach used to assess how cumulative epigenetic deregulation compromises ERN robustness in malignant cells. (**B**) Log_2_-ratio of peptide L/H ratio relative to the mean value of parental WT HCEC cells for the indicated differentially modified histone peptides in transformed lines. Three biological replicates per condition are shown. One, two, three, and four asterisks: *P* < .05, *P* < .05, *P* < .001, and .0001, respectively. Gray cross: Undetected peptides. (**C**) Fitness scores of ERG-KO populations in parental WT and transformed KP-C or KP-A HCEC cells. Values: mean ± SEM of three biological replicates. Vertical drop from line of identity indicates synthetic sick/lethal phenotypes. (**D**, **E**) Fitness scores of ERG-KO populations in the indicated HCEC lines. Values: mean ± SEM of three biological replicates. Black and red gene names: enhanced or KP-unique synthetic phenotypes, respectively, based on linear regression analysis in the two conditions. Representative interactions are shown; the full list is in Supplementary Fig. S6F and Supplementary Table S4.

While combinatorial genetic perturbation sensitively revealed inter- and intra-class functional interactions within the ERN, disease-free somatic cells effectively tolerated loss of multiple components and remained highly robust to interference. We therefore asked whether additional perturbation of the epigenome via signaling-mediated processes would synergize with genetic inactivation of ERN components. To this end, we induced neoplastic transformation of WT, *ARID1A*-, and *CREBBP*-KO HCEC cells via inactivation of p53 and expression of oncogenic KRAS^G12C^ (KP), common drivers of colon cancer (Fig. 4A and Supplementary Fig. S6A–D).

All populations responded to oncogene activation as expected, increasing proliferation rate and acquiring clonogenic ability in semi-solid medium (Supplementary Fig. S6C and D). Interestingly, the moderately impaired fitness of *ARIDA1*-KO cells was rescued upon transformation, with cells of all genotypes displaying comparable proliferation rates (Supplementary Fig. S6C). In line with the notion that activation of oncogenic signaling affects the cell epigenome, we detected widespread changes in histone modifications by MS across all transformed lines (Fig. 4B and Supplementary Table S3). Taking advantage of the modular and isogenic nature of the system, we examined whether the combined perturbation (absence of one ERN component together with oncogene-induced chromatin alterations) impaired the ERN resilience to further disruption (Fig. 4A). Once again, to minimize technical variability and enable direct comparison of the results, we performed the large-scale fitness assay in parallel in the three genetic backgrounds. Inactivation of ERGs in KP-*CREBBP*-KO cells (KP-C-DKO populations) had a mild global effect, with most populations exhibiting negligible changes in cell fitness (Fig. 4C and D). Thus, in agreement with the results from untransformed cells, the absence of CREBBP is effectively buffered even when oncogenic signaling alters homeostatic levels of many histone modifications. Importantly, the bias toward KAT-related synthetic phenotypes was maintained, and all functional compensators identified in untransformed cells were detected upon transformation as well (Fig. 4D and Supplementary Fig. S6E and F). The extent of fitness reduction was greater, indicating partial ERN destabilization in transformed cells, but the network was far from collapsing and largely maintained its functionality even after a triple perturbation (Fig. 4D). Notably, oncogene-induced chromatin changes made CREBBP-deficient cells less sensitive to EP300 loss, and more dependent on structurally unrelated KATs (Supplementary Fig. S6E).

In contrast, inactivation of individual ERGs in KP-*ARID1A*-KO cells (KP-A-DKO) revealed a striking fragility of the ERN across all classes: 99 populations showed synthetic-lethal phenotypes, 34 of which exhibited further decreased fitness compared with *ARID1A*-KO untransformed cells, whereas 65 phenotypes were uniquely detected upon transformation (Fig. 4C and E; Supplementary Table S2; Supplementary Fig. S6E and F—blue dots: enhanced phenotype, red dots: unique interactions; Supplementary Table S4). Transformation-specific phenotypes revealed interactions between ARID1A and regulators of DNA methylation, which were not detected in untransformed cells, highlighting that unexpected interdependencies between parallel pathways are exposed by oncogene-induced epigenetic disruption (Supplementary Fig. S6F). Notably, *ARID1B* was amongst the strongest dependencies of KP-A-DKO cells, but it was not an outlier (Fig. 4E), once again suggesting that duplicated genes represent only one of multiple layers of functional compensation contributing to ERN robustness, and that all these layers are destabilized by oncogenic signaling. The large effect size detected in multiple KP-A-DKO populations, suggestive of network collapse, was specific to transformed cells and was not due the presence of the oncogenic drivers per se, as we observed only mild effects in both KP-WT-DKO cells and KP-C-DKO (Supplementary Fig. S6G and H, Fig. 4C and D, Supplementary Table S2). Thus, epigenetic alterations induced by oncogenic signaling do not substantially affect the ERN robustness on their own, but can induce synthetic fragility when ARID1A is concomitantly inactivated. Given the high prevalence of *ARID1A* inactivating mutations across cancer types (Supplementary Fig. S4C and D), this broad and cancer-specific ERN fragility is of high clinical significance.

### Functional compensation among KATs and degenerate histone acetylation ensure robust epigenetic regulation

**Figure 5.**
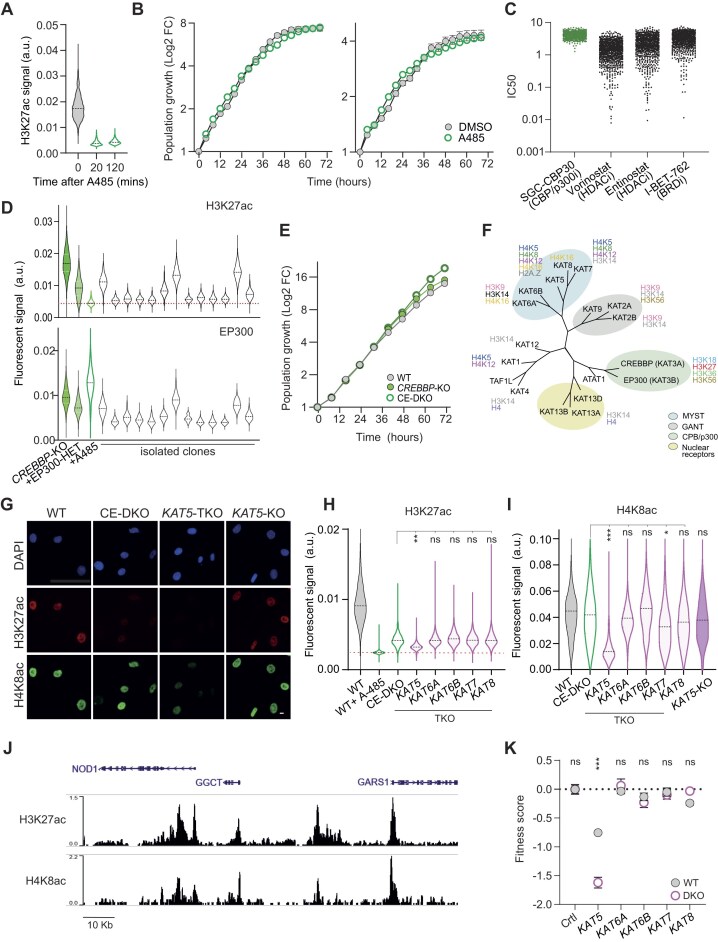
Cooperativity amongst HATs renders CREBBP/EP300 dispensable in human somatic cells. (**A**) Quantitative immunofluorescence microscopy of HCECs treated with 4 μM A-485. Distributions of H3K27ac signal intensity in single cells at the indicated timepoints are compared. n = 1206–1376, two-tailed *t*-test *P*-values < .0001. (**B**) Proliferation curves of HCEC and HME1 cell populations during treatment with DMSO or 4 μM A-485, refreshed every 12 h. Values are the increase in confluence relative to the first timepoint, expressed as mean ± SEM of five biological replicates. *T*-test comparing the slope of a linear model fitted to the log_2_-transformed values between 24 and 48 h, one asterisk: two-tailed *P* < .05. (**C**) Sensitivity data of cell lines across cancer types for the indicated drugs from the Sanger GDSC2 database. HDACi Vorinostat and Entinostat and BRD4i I-BET-762 are shown as a reference. Data for the CREBBP/EP300i SGC-CBP30 are shown as A-485 was not tested. 716 < N < 959 cancer cell lines. (**D**) Quantitative immunofluorescence microscopy of representative monoclonal *CREBBP*-KO HME1 populations treated with sgRNAs targeting *EP300*. Distributions of H3K27ac and EP300 signal intensity in single cells are compared with parental *CREBBP*-KO cells with WT *EP300* alleles, a clone of *CREBBP*-KO cells with an inactivate allele (+EP300-HET) and *CREBBP*-KO cells treated with CREBBP/EP300i (+A485). Clones were scored as KO if the mean H3K27ac signal was within mean ± 2SD of the A485-treated population, shown as red dotted line. 77 < N < 1243 nuclei per condition. (**E**) Proliferation curves of HME1 cell populations of the indicated genotypes. Values are the increase in cell count relative to the first timepoint, expressed as mean ± SEM of six biological replicates. T-test comparing the slope of a linear model fitted to the log_2_-transformed values between 24 and 48 h, four asterisks: two-tailed *P* < .0001. (**F**) Phylogenetic tree of the KAT enzymes. Proteins are grouped by families and their major histone substrates are indicated. Adapted from Di Cerbo and Schneider [85]. Quantitative immunofluorescence microscopy of the indicated HME1 lines assessing the indicated acetylated residues. Representative images (**G**) and quantification of the fluorescent signal in single cells (**H**, **I**). Scale bar: 10 μm, 899 < *n* < 6855, two-way ANOVA with Dunnett’s multiple comparisons test; one, two, or three asterisks: *P* < .05, .01, or .001, respectively. (**J**) ChIP-seq tracks of the indicated histone acetylation marks profiled in human mammary epithelial cells MCF10A, retrieved from Cistrome Data Browser. (**K**) Fitness scores of either WT or CE-DKO HME1 cells 96 h after transfection with the indicated KAT-targeting gRNAs. Values, mean ± SEM of three replicates. Unpaired two-tailed *t*-test; three asterisks: *P* < .001.

Based on the observations that (i) CREBBP loss is fully buffered both in HCEC and HME1 cells (Fig. 2A), (ii) histone marks acetylated by CREBBP are only mildly affected (H3K27ac) or unaltered (H3K18ac, H3K36ac) in its absence (Fig. 2C–G), (iii) combined loss of CREBBP and EP300 only partly affects network functionality (Fig. 2I), and (iv) combined inactivation of other KATs shows synthetic-sick phenotypes of comparable or even greater magnitude than *EP300* knockout (Fig. 3F and Supplementary Fig. S6E), we hypothesized that an intra-class functional network of KATs remodels upon perturbation to ensure maintenance of epigenetic regulation by histone acetylation. To investigate, we treated HCECs and HME1s with A485, a selective catalytic inhibitor of CREBBP and EP300 [69] that leads to rapid loss of H3K27ac 20 min after treatment (Fig. 5A).

Over the course of 3 days, the fitness of A485-treated cells was comparable to that of control cells, further suggesting that combined loss of the paralogue pair activity is effectively buffered within the ERN (Fig. 5B). Our results match patterns observed in large-scale drug-sensitivity studies, which have found many cancer cell lines to be insensitive to CREBBP/EP300 inhibitors (Fig. 5C). Since short-term fitness assays may miss or underestimate long-term deleterious effects associated with compromised ERN functionality, we challenged the system further and asked whether we could derive and maintain monoclonal populations lacking both CREBBP and EP300. Starting from the characterized *CREBBP*-KO HME1 population, we inactivated *EP300*, isolated single cells, let them grow for 20 days, and assessed EP300 and H3K27ac levels by quantitative immunofluorescence. Out of 60 tested clones, 44 displayed background levels of EP300 and DNA sequencing of selected populations confirmed gene knock-out (Fig. 5D); thus, the paralogue pair is dispensable for both cell viability and maintenance of long-term proliferative capacity. Interestingly, H3K27ac levels in CE-DKO clones were strongly decreased, but not as low as those measured in A485-treated cells, indicating that CE-DKO cells maintain minimal levels of H3K27ac even in the absence of CREBBP/EP300. Twenty days after clone isolation, CE-DKO cells were as fit as WT or *CREBBP*-KO cells, suggesting that the deleterious effects observed shortly after inactivation of the paralogue pair (Fig. 2I) were transient, and that cells eventually adapted to their loss (Fig. 5E).

To begin to uncover how cells remodel the ERN and adapt to the loss of two major chromatin regulators, we asked whether other KATs may sustain basal H3K27ac levels in CE-DKO cells. To do so, we selected 5 KATs whose loss induced synthetic-sick phenotypes in CREBBP-KO cells (Fig. 3F and Supplementary Fig. S6E) and inactivated the corresponding genes in CE-DKO cells, generating triple-knockout (TKO) cells. Interestingly, all KATs that exhibited functional interactions with CREBBP belong to the MYST family and exert partly redundant functions at various histone residues (Fig. 5F). While inactivation of KAT6A/B, KAT7, and KAT8 in TKO cells did not affect H3K27ac level, loss of KAT5 significantly decreased the mark to levels close, yet not identical, to those measured in A485-treated cells (Fig. 5G and H). Furthermore, CREBBP/EP300/KAT5 TKO cells, but not cells only lacking KAT5, also exhibited strongly reduced levels of H4K8ac, indicating that the three KATs cooperate in maintaining homeostatic levels of both H3K27ac and H4K8ac (Fig. 5G and I). Notably, genome wide patterns of both histone marks are highly correlated, as both are enriched at active promoters and active enhancers (Fig. 5J). Thus, while the presence of KAT5 may only minimally compensate for the loss of CREBBP/EP300 with respect to H3K27ac, its ability to fully maintain H4K8ac at regulatory elements may allow cells to preserve transcriptional activity and overall fitness. Indeed, fitness assays revealed a strong synthetic sick phenotype in CREBBP/EP300/KAT5 TKO cells as early as 72 h after KO, demonstrating a cooperation among the three KATs in sustaining ERN functionality and epigenetic regulation via degenerate histone acetylation (Fig. 5K).

## Discussion

In this study, we sought to reveal how functional interactions among diverse epigenetic regulators and emergent system-level properties bolster robustness in the human ERN. We find that the presence of multiple compensatory mechanisms makes normal somatic cells remarkably resilient to both mutational and signaling-related disruption of epigenetic regulation, but that context-specific vulnerabilities are exposed as the epigenome is progressively deregulated in disease.

Although the mere assessment of cell fitness as a readout of epigenetic dysregulation has limitations (see dedicated section below), the detection of synthetic sick phenotypes in the double KO populations or in transformed cells demonstrates that the assay can sensitively capture altered ERN output when buffering mechanisms are disabled: dispensable components as defined in single KO populations, can become indispensable depending on context. Inspired by epistasis studies in yeast [10], this approach enabled a systematic interrogation of ERN components in human somatic cells using an isogenic system, and sensitive identification of functional interactions in the absence of confounding effects. While the loss of individual ERN components may have stronger effects in more complex settings, as suggested by the embryonic lethality of some murine knock-out models [70, 71], the principles that we uncovered using cells from two distinct lineages likely extend to multiple adult tissues. Interestingly, ERN robustness appears to be stage-dependent: several ERGs have been shown to be required for embryonic development but dispensable for adult tissue homeostasis, suggesting that maintenance of epigenetic memory and cell identity is more robust than establishment of *de novo* epigenetic states [72–75].

By characterizing ARID1A- and CREBBP-deficient cells, we find that functional compensation is provided by multiple layers of redundancy, which include, but are not limited to, duplicated components encoded by paralogue genes. In both cases, combined loss of the paralogue pair destabilizes the ERN, but it is not enough to induce network collapse, highlighting the existence of additional back-up mechanisms. Interestingly, robustness arises through distinct modalities. Numerous components across the entire ERN interact functionally with ARID1A/B (inter-class cooperativity), leading cells to become sensitized to further perturbation of regulators from across these diverse functional classes. In contrast, the collective activity of multiple histone acetyltransferases forms a specific subnetwork that supports ERN functionality via degenerate histone acetylation (intra-class cooperativity). Supporting our findings, mice lacking both ARID1A and ARID1B in liver and skin are viable and reach adulthood [18]. This is partly attributed to rewired activity of structurally distinct cBAF or PBAF complexes and, in part, though SWI/SNF-independent mechanisms. Moreover, the observation that individual acetylation residues, including H3K27ac, are dispensable for active transcription and their loss minimally impacts steady-state gene-expression programs [39, 76], further underscores the idea that distinct acetylated histone marks might exert partly redundant functions.

In line with the notion that epistatic mutational phenotypes are underpinned by a range of molecular mechanisms and do not necessarily involve physical interaction between two proteins [77], the size of physical interaction networks did not correlate with the number of detected genetic interactions, and in fact loss of CREBBP, which is among the most highly connected ERN components based on protein–protein interactions [78], destabilized the network only minimally; conversely, most ARID1A functional interactors have not been reported to physically bind to it. Thus, functional assays that go beyond detection of biochemical interactions are required to map synergistic activities within the ERN and dissect system-level mechanisms of genome regulation.

Our findings have important implications for disease, particularly cancer, where mutations in ERGs are common. Seemingly paradoxical is the observation that loss of ARID1A mildly compromises cell fitness in normal cells, but inactivating *ARID1A* mutations are broadly selected across cancers [79]. Interestingly, experimental transformation of HCEC cells abolished the fitness defect of ARID1A-decient cells, showing that ARID1A becomes fully dispensable in malignant cells. *ARID1A* and *ARID1B* mutations co-occur in patients from multiple cancer types and >30% of *ARID1A*-mutant cell lines harbor *ARID1B*-inactivating mutations [17, 18]; thus, even the combined loss of the paralogue pair is tolerated, and potentially beneficial to cancer cells. Remarkably, while malignant cells do not require ARID1A to keep proliferating, its loss creates a strong synthetic fragility in the ERN, whereby loss of a second epigenetic regulator, combined with epigenetic alterations induced by oncogenic signaling, in many cases leads to network collapse. Our findings uncover many opportunities for therapeutic interventions in ARID1A-deficient cancers, which represent ∼8% of cases across all malignancies [79]. The observed synthetic fragility is of particular importance considering that *ARID1A* mutations also accumulate in normal tissues; in the absence of activated oncogenes, the ERN of nontransformed cells would remain largely insensitive to a potential pharmacological perturbation, minimizing deleterious effects in normal tissues. Furthermore, we speculate that the broad functional connectivity of ARID1A may also relate to its driver role in cancer: network components with a high number of genetic interactions have been described as “buffers” or “capacitors” if their activity controls the exposure of knockout phenotypes [80]. In an analogy with HSP90, which buffers cryptic phenotypic variation at the protein level and whose loss promotes selection of favorable morphological traits during species evolution, it is conceivable that destabilization of the ERN in ARID1A-deficient cells may expose favorable, nonphysiological cellular states that can be selected during cancer evolution. Supporting this scenario, chromatin regulatory hubs have been identified as capacitors in yeast and *Caenorhabditis elegans*, indicating that the ERN exerts control over phenotypic variation [81, 82]. Our findings also uncover vulnerabilities of CREBBP-deficient cancers: even though oncogenic signaling does not induce a general destabilization of the ERN in this context, we observed a “local” synthetic fragility of the HAT subnetwork, which suggests that combinatorial targeting of multiple HATs may have deleterious effects on cancer cells while sparing their normal counterparts.

Limitations of the study: Fitness is a broad phenotype that emerges from various underlying molecular phenomena within a cell. Measuring a single, common parameter is essential when looking for epistatic phenotypes between functional classes with distinct molecular functions. However, there is not a strict linear relationship between altered epigenetic regulation and cellular fitness, and some functional relationships may remain unrevealed by this approach. While we have uncovered several interactions across the ERN, how disruption of epigenetic control impacts cell fitness remains to be elucidated. The diversity of epigenetic functions means that diverse molecular mechanisms likely underpin the observed effects, some of which may act at timescales beyond the length of our assay. Finally, while we have shown efficient editing of both dispensable and indispensable genes (Supplementary Fig. S1E–H), as for any large-scale knockout assay, off-target-induced false positives and false negatives due to inefficient editing cannot be excluded.

In conclusion, our findings highlight the importance of degenerate pathways within and across epigenetic regulatory layers in sustaining a robust ERN in normal somatic cells. Degeneracy is observed at the level of individual modifiers that share substrates with other enzymes, distinct protein subcomplexes of similar molecular function but divergent roles, histone modifications co-occupying common genomic regions, and parallel pathways converging on a common output. Since some of these back-up mechanisms are disrupted in cancer, weaknesses in the ERN are exposed and offer opportunities to interfere with the disease.

## Supplementary Material

gkaf297_Supplemental_Files

## Data Availability

ChIP-seq and RNA-seq data have been deposited at NCBI-GEO (SuperSeries GSE277002). Histone mass-spectrometry data have been deposited at ProteomeXchange Consortium (PXD039819).
