# Supplementary material for: Systematic genetic perturbation reveals principles underpinning robustness of the epigenetic regulatory network
Source: Nucleic Acids Res. 2025 Apr 17;53(7):gkaf297. doi: 10.1093/nar/gkaf297 (PMC12000879; doi:10.1093/nar/gkaf297)
Supplement: gkaf297_Supplemental_Files [file gkaf297_supplemental_files.zip › Suppl. Figures.pdf]

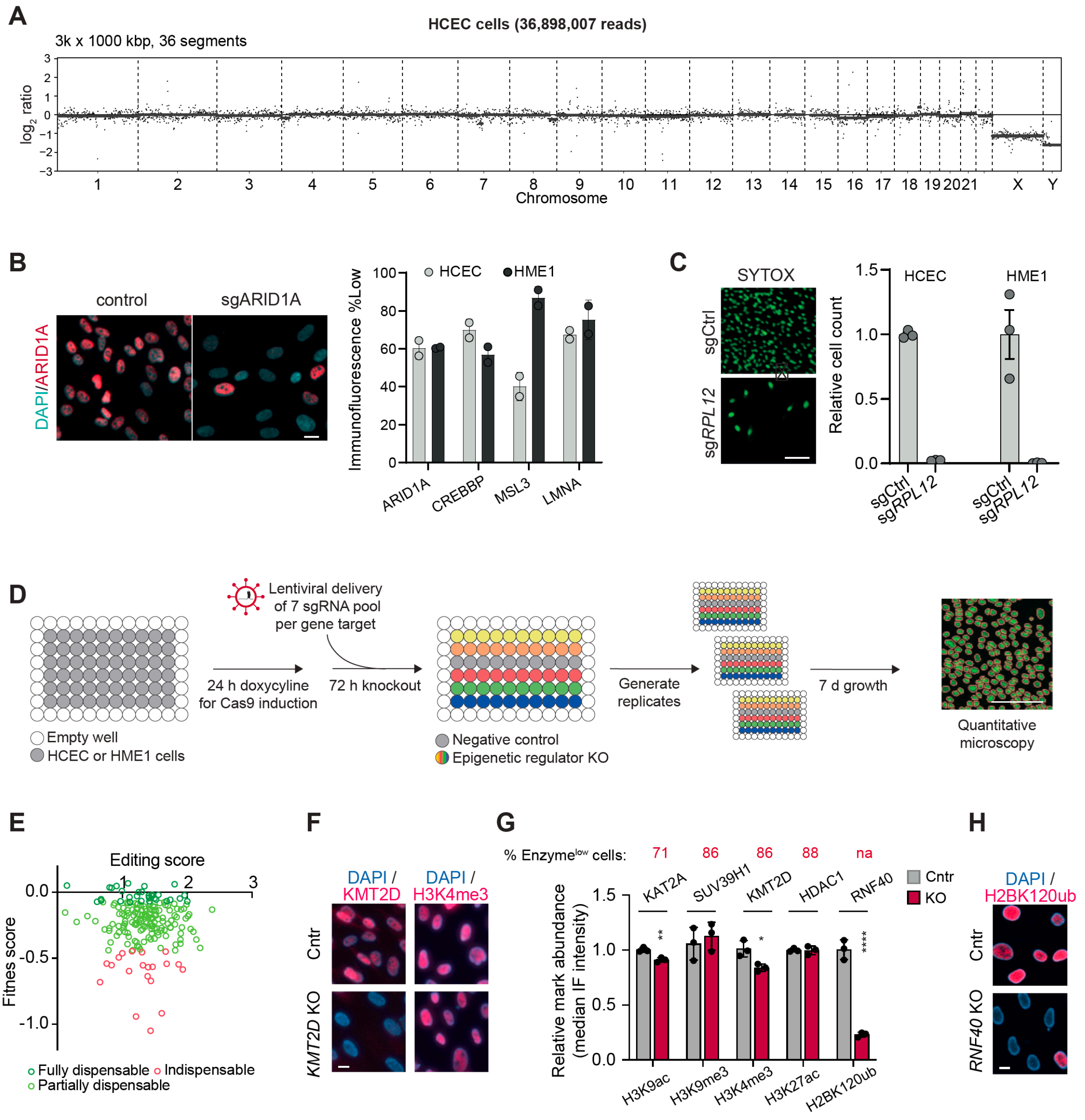

Figure S1. **Efficient generation of epigenetically-disrupted cells.**

A. Karyotype analysis of HCEC cells, assessed by low-pass whole-genome sequencing. The abundance of each genomic segment relative to the rest of the genome is plotted. **B**. Quantitative immunofluorescence microscopy of HCEC and HME1 cells transduced with gRNAs targeting the indicated genes. Representative images and quantification of cells with intensity values lower than the 5th percentile of the negative control distribution. Scale bar: 10 μm. **C**. Fitness assay of HCEC and HME1 cells transduced with the indicated sgRNAs. Representative images and quantification of cell count after 11 d of growth relative to the negative control. Scale bar: 100 μm. **D**. Schematic of the experimental pipeline used in the study. **E.** Relationship between fitness scores of populations with inactivated ERGs and the Editing score of the corresponding sgRNA (on-target activity), as determined in Ref. (41). **F**, **H**. Immunofluorescence microscopy of HCEC cells upon KMT2D (**F**) or RNF40 (**H**) KO using the indicated antibodies. KMT2D loss does not substantially alter H3K4me3 levels, whereas RNF40-KO cells lose H3K120Ub.**G.** Quantification of global levels for the indicated histone marks in cells lacking the corresponding histone writer or eraser (gene name at the top), as assessed by quantitative immunofluorescence. Values are the median value across cells in three replicate wells. One, two or four asterisks: $p < 0.05, 0.01, 0.0001$, respectively (two-tailed Student's t-test). The percentage of edited cells in each population is indicated in red (not quantified for RNF40, due to lack of suitable antibodies). Inactivation of dispensable genes (KAT2A, SUV39H1, KMT2D and HDAC1) or indispensable genes (RNF40) have differential impact of cognate histone marks.

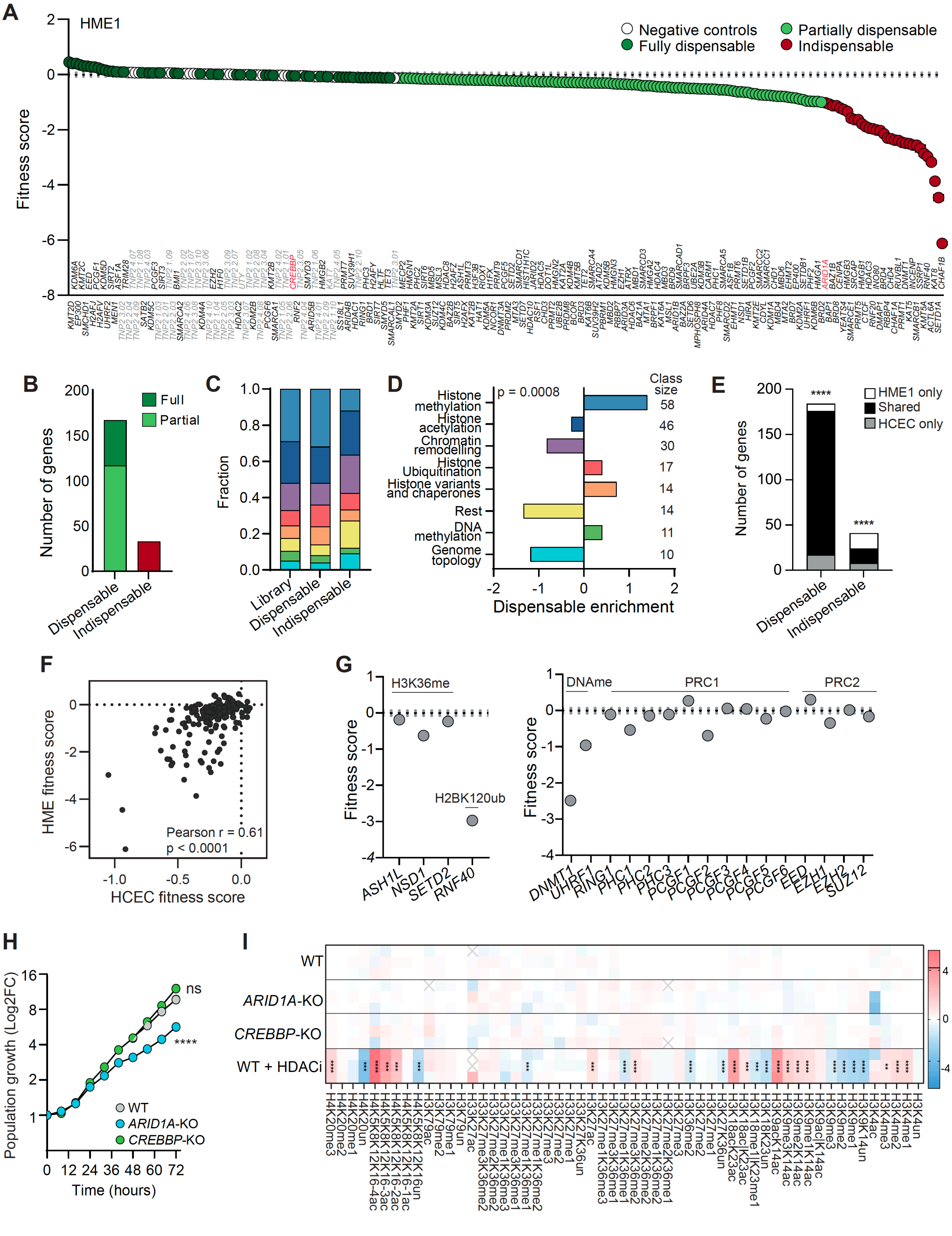

Figure S2. **Large-scale fitness assay in HME1s.**

A. Profile of fitness scores in HMEs. Values: mean ± SEM of 3 replicates. Black and grey dotted lines: mean and minimum/maximum negative control values, respectively.**B**. Classification of ERGs in HMEs based on results in **A**. **C-D**. Fraction (**C**) and enrichment (**D**) of fully dispensable or indispensable ERGs across functional class. Values in D are the $\log_2$ ratio of observed vs expected dispensable proportion. Statistical significance of the enrichment (two-tailed χ^2 test) and the size of each functional class are indicated. **E**. Overlap of dispensable and indispensable genes identified in HCEC or HME1 cells. Four asterisks: $p < 0.0001$, hypergeometric test. **F**. Correlation

between fitness scores of ERG knock-out populations in HCEC and HME1 cells. **G**. Fitness scores for HME1 populations with the indicated genes knocked-out. **H**. Proliferation curve of the indicated HME1 isogenic lines. Values: mean ± SEM of 6 replicates. P-value. T-test comparing the slope of a linear model fitted to the log_2-transformed values between 24 and 48 hours, four asterisks: two-tailed $p < 0.0001$. **I**. Log_2-ratio of L/H ratio relative to the mean value of WT cells for the indicated differentially modified histone peptides in the isogenic HME1 lines, and WT cells treated with 50 nM Quisinostat (HDACi). Three biological replicates per condition are shown. One, two, three and four asterisks: $p < 0.05$, $p < 0.01$, $p < 0.001$ and 0.0001, respectively, for differential peptides. Grey cross: undetected peptides.

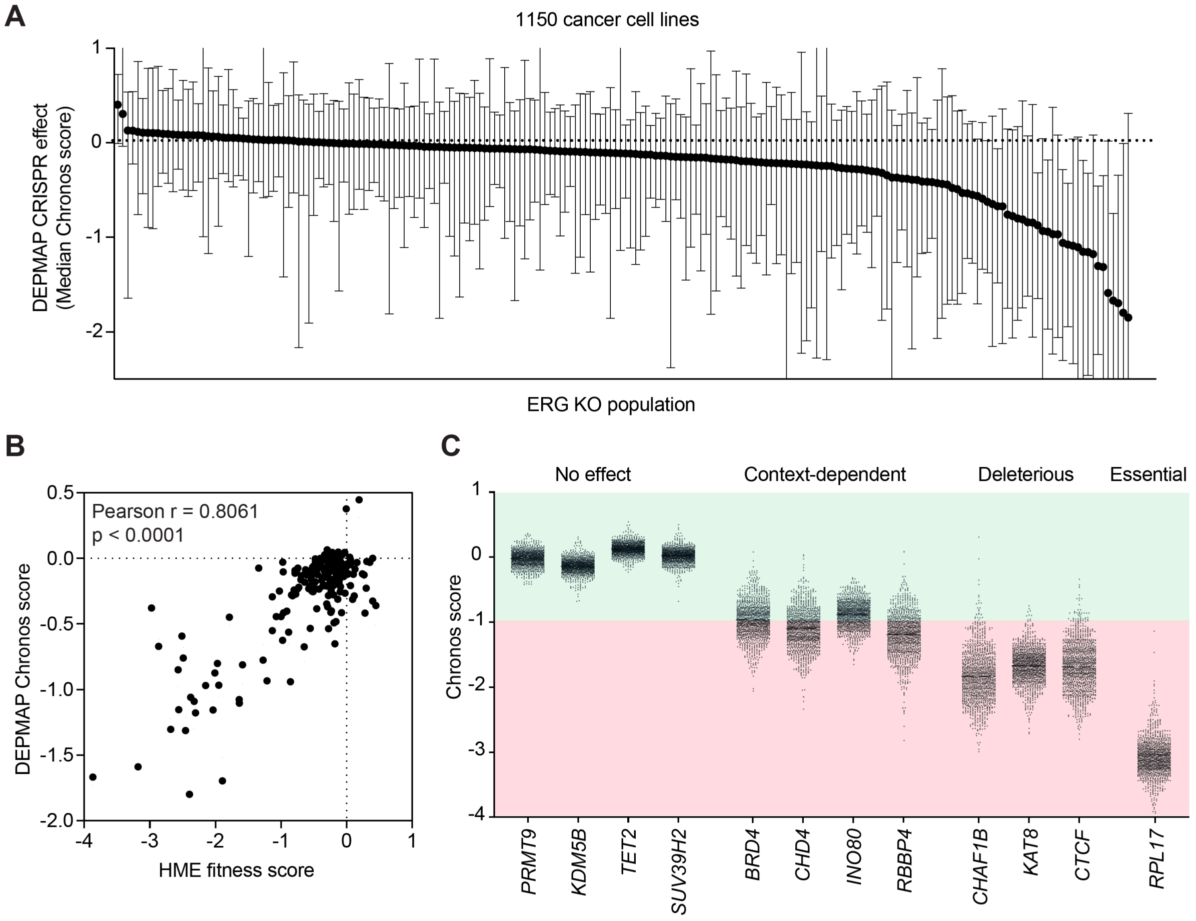

Figure S3. Context-specific effects confound the analysis of ERG essentiality in cancer cells.

A. Profile of median fitness values across 1150 cancer cells lines upon inactivation of individual ERGs as measured by the DEPMAP project. Values: median Chronos score ± the range across all lines. As per DEPMAP criteria, a gene is defined as essential if the corresponding chronos score is < -1. **B**. Correlation between fitness scores of ERG knock-out populations in HME1 cells and across cancer cells lines profiled in DEPMAP (median value across lines). **C**. Distribution of Chronos scores across cancer cells lines upon KO of the indicated genes. Green and red indicates cell lines where genes would be considered dispensable or required for maintenance of cell fitness, respectively. The ribosomal gene RPL17 is shown as an example of essential genes.

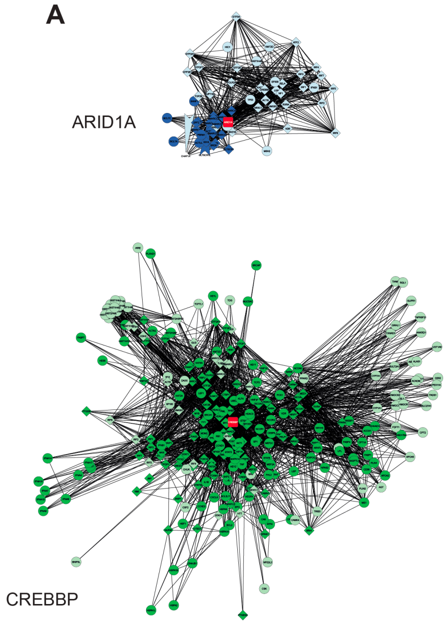

B

NCG 7.0	ARID1A	CREBBP
Gene Expression in tissues	100 .00% (43/43)	100.00 % (43/43)
Protein expression in tissues	91.11 % (41/45)	100.00 (45/45)
Protein interactions	94	425
miRNA interactions	101	47
Tolerance to LOF (median = 0.885)	0.071	0.066

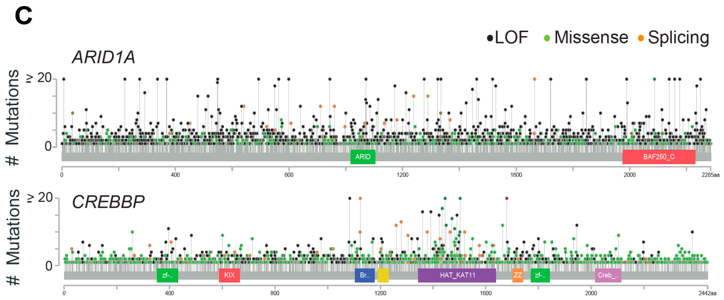

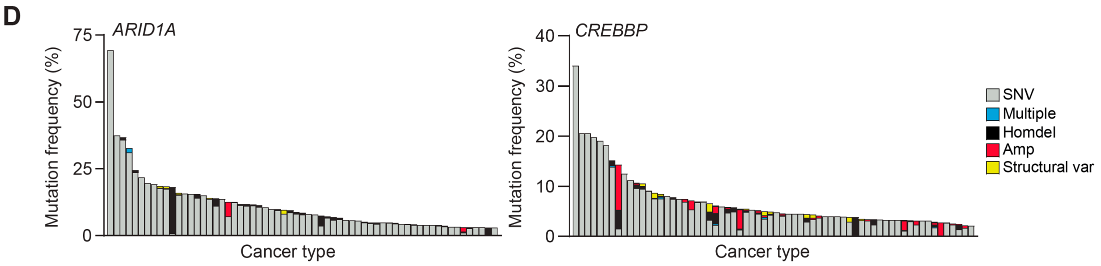

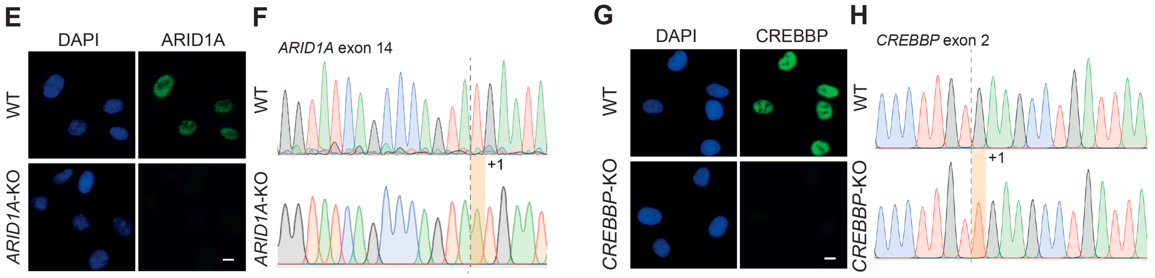

Figure S4. *ARID1A* and *CREBBP* mutations in disease.

A. Visualization of ARID1A and CREBBP interactions as retrieved from the STRING database (86). **B**. Summary information about *ARID1A* and *CREBBP* from the Network of Cancer Genes database (87). **C**. Visualization of mutations detected in *ARID1A* and *CREBBP* across cancer type as retrieved from cBioPortal. **D**. Quantification of single nucleotide variants (snv) and other genomic alterations in individual cancer types as retrieved from cBioPortal. **E, G**. Immunofluorescence microscopy confirming loss of the indicated proteins in the isolated knock-out HCEC/HME1 populations. Scale bar: 10 µm. **F, H**. Traces of Sanger sequencing confirming biallelic inactivation of the indicated genes in isolated clonal lines.

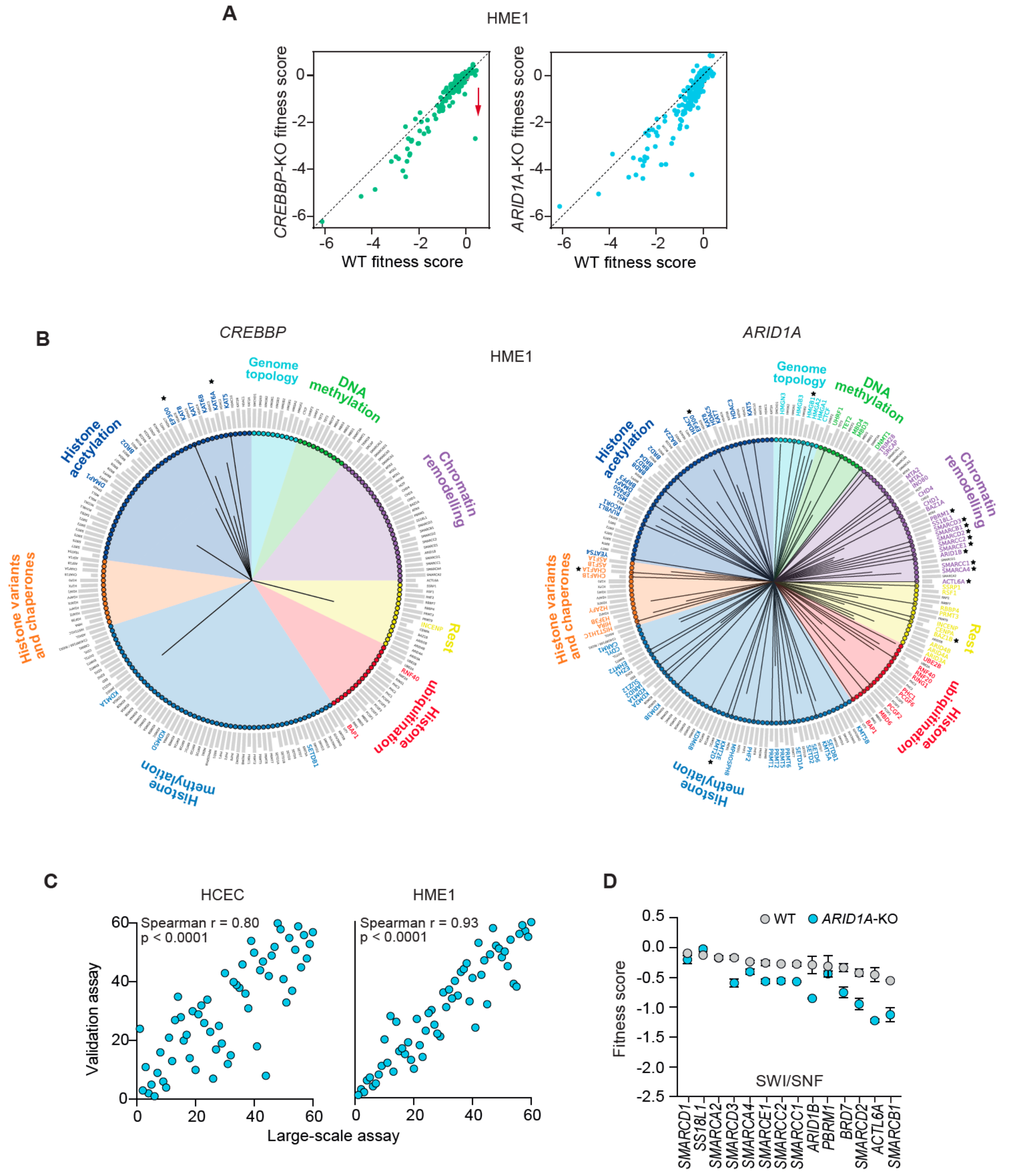

Figure S5. **Functional interactions of *ARID1A* and *CREBBP* in HME1 cells.**

A. Fitness scores of ERG-KO populations in WT and *CREBBP*-KO or *ARID1A*-KO HME1 cells. Values: mean ± SEM of three biological replicates. Vertical drop from line of identity (red arrow) indicates synthetic sick phenotypes. **B.** Identified CREBBP or ARID1A functional interactions with ERN components in HME1 cells arranged by functional class. Edges scaled according to interaction strength estimate up to maximum estimate value of 1. Grey bar, fitness score in WT cells. Black star: physical interactor by STRING database. **C.** Correlation between the fitness of selected KO populations measured in the large-scale assays and in validation experiments (gene rank). The r and p values of the Spearman correlation is indicated. **D**. Fitness scores of SWI/SNF complex subunits in WT and *ARID1A*-KO HCEC cells.

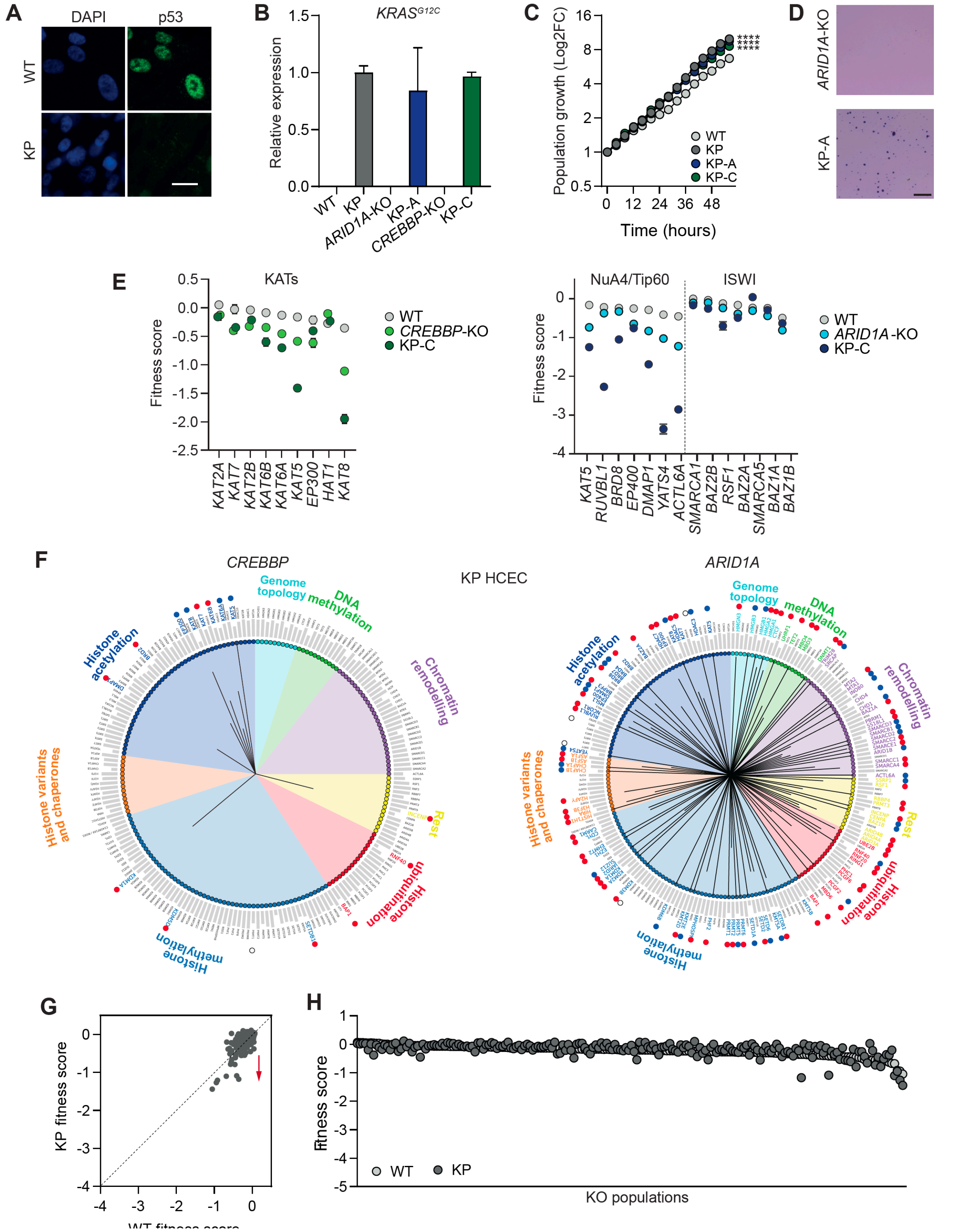

Figure S6. **Neoplastic transformation does not alter ERN robustness on its own.**

A. Immunofluorescence microscopy confirming loss of p53 in transformed HCEC cells. Scale bar: 10 µm. **B**. Detection of exogenous *KRAS*^{G12C} by RT-qPCR in the indicated HCEC lines. ddCT values are expressed as relative to KP cells, mean ± SEM of 3 replicates.**C**. Proliferation curve of the indicated HCEC isogenic lines. Values: mean ± SEM of 6 replicates. T-test comparing the slope of a linear model fitted to the log_2-transformed values between 24 and 48 hours, three, four asterisks: two-tailed $p < 0.001$ or 0.0001, respectively. **D**. Representative images of colonies grown in soft agar. Similar results were obtained with KP and KP-C cells. scalebar = 3 mm. **E**. Fitness

scores of the indicated KO populations in the isogenic HCEC lines. Values: mean ± SEM of three replicates. **F**. Identified CREEBP or *ARID1A* functional interactions with ERN components in transformed HCEC cells arranged by functional class. Edges scaled according to interaction strength estimate up to maximum estimate value of 1. Grey bar, fitness score in WT cells. Black star: physical interactor by STRING database. **G, H**. Fitness scores of ERG-KO populations in untransformed WT and KP HCEC cells. Values: mean ± SEM of three biological replicates. Vertical drop from line of identity in **G** (red arrow) indicates synthetic sick phenotypes.

Supplementary Reference

86 Szklarczyk D, Kirsch R, Koutrouli M *et al.* The STRING database in 2023: protein–protein association networks and functional enrichment analyses for any sequenced genome of interest. *Nucleic Acids Res* 2023; 51 :D638–46. https:// doi.org/ 10.1093/ nar/ gkac1000

87 Dressler L, Bortolomeazzi M, Keddar MR *et al.* Comparative assessment of genes driving cancer and somatic evolution in non-cancer tissues: an update of the Network of Cancer Genes (NCG) resource. *Genome Biol* 2022; 23 :35. https:// doi.org/ 10.1186/ s13059- 022-02607- z
